# Exploration of newly synthesized azo-thiohydantoins as the potential alkaline phosphatase inhibitors via advanced biochemical characterization and molecular modeling approaches

**DOI:** 10.1186/s13065-024-01149-8

**Published:** 2024-03-06

**Authors:** Hafiz Muhammad Attaullah, Syeda Abida Ejaz, Pervaiz Ali Channar, Aamer Saeed, Rabail Ujan, Seema Zargar, Sajid Ali Channar, Reshma Sahito, Tanveer A. Wani, Qamar Abbas

**Affiliations:** 1 Department of Pharmaceutical Chemistry, Faculty of Pharmacy, The Islamia University of Bahawalpur, Bahawalpur, 63100 Pakistan; 2 Department of Basic Sciences and Humanities, Faculty of Information Science and Humanities, Dawood University of Engineering and Technology, Karachi, 74800 Pakistan; 3 Department of Chemistry, Quaid-I-Azam University, Islamabad, 45320 Pakistan; 4 Dr. M. A. Kazi Institute of Chemistry, University of Sindh, Jamshoro, Pakistan; 5 Department of Biochemistry, College of Science, King Saud University, P.O.Box 22452, Riyadh, 11451 Saudi Arabia; 6 Department of Zoology, University of Sindh, Jamshoro, Pakistan; 7 Department of Pharmaceutical Chemistry, College of Pharmacy, King Saud University, P.O.Box 2457, Riyadh, 11451 Saudi Arabia; 8 Department of Biology, College of Science, University of Bahrain, Sakhir, 32038 Kingdom of Bahrain; 9 College of Natural Sciences, Department of Biological Sciences, Kongju National University, Gongju, 32588 Republic of Korea

**Keywords:** Azo, Azo-thiohydantoins, Molecular docking, MD simulations, Alkaline phosphatase

## Abstract

In the current study, Azo-Thiohydantoins derivatives were synthesized and characterized by using various spectroscopic techniques including FTIR, ^1^H-NMR, ^13^C-NMR, elemental and HRMS analysis. The compounds were evaluated for alkaline phosphatase activity and it was observed that among all the synthesized compounds, derivative **7e** exhibited substantial inhibitory activity (IC_50_ = 0.308 ± 0.065 µM), surpassing the standard inhibitor (L–Phenyl alanine, IC_50_ = 80.2 ± 1.1 µM). Along with this, these derivatives were comprehensively examined regarding the electronic properties and reactivity of the synthesized compounds using Density Functional Theory (DFT) calculations, where the results were found very promising and the synthesized compound were found stable. After that, SwissADME evaluations highlighted compounds for their favorable physicochemical properties, including solubility and drug-likeness. Molecular docking exhibited the strong binding affinities of **7f** and **7e** derivatives with intestinal alkaline phosphatase (IAP), further supported by Molecular Dynamics (MD) simulations. This comprehensive integration of experimental and computational approaches sheds the light on the potential therapeutic applications of the synthesized compounds. By providing a detailed investigation of these aspects, this research opens the avenues for the development of novel pharmacologically active compounds with diverse applications.

**Supplementary Information:**

The online version contains supplementary material available at 10.1186/s13065-024-01149-8.

## Introduction

Alkaline phosphatase (ALP) is a group of isoenzymes that catalyze the hydrolysis of phosphate monoesters at basic pH [1]. It is an ubiquitous membrane-bound glycoprotein located on the outer layer of the cell membrane [2]. ALP is found in various tissues throughout the human body, including the liver, bone, placenta, kidneys, and intestines. The enzyme is involved in several biological processes, including bone mineralization, cellular signal transmission, and phospholipid metabolism. The reference intervals for ALP levels in the blood of adults and females at 37 °C can be found in clinical chemistry reference ranges [3]. ALP tests are commonly used to diagnose liver damage or bone disorders.

Among the types of ALP, the tissue-nonspecific alkaline phosphatase (TNAP) is an enzyme that is found in various tissues throughout the human body [4], including the liver, bone, placenta, kidneys, and intestines. It plays a significant role in regulating mineralization of bone tissue, as well as phospholipid metabolism and cellular signal transmission. Any mutation in the TNAP gene can lead to a rare disorder called hypophosphatasia [5], which is characterized by poor mineralization of bones and teeth, as well as other health complications. Additionally, TNAP is a marker for liver damage, and ALP tests are commonly used to assess liver function. TNAP has also been implicated in regulating purinergic signaling, which plays a role in immune response and inflammation.

Another type i.e., intestinal alkaline phosphatase (IAP) is a brush-border enzyme that is expressed throughout the gastrointestinal tract and is involved in several biological processes [6], including gut mucosal defense, regulation of gut homeostasis, and modulation of inflammatory mediators. IAP is secreted by enterocytes and is present on the apical surface [7]. It plays an important role in detoxifying bacterial lipopolysaccharides (LPS), which are the major components of the bacterial cell wall and can cause inflammation and tissue damage. IAP has been shown to be decreased during conditions that commonly affect the gut [8], i.e. inflammatory bowel disease (IBD) and type-2 diabetes. It appears that IAP supplementation may have therapeutic potential in the treatment of these conditions by reducing inflammation and protecting the gut barrier [9]. In addition, evidence to reveal that IAP may play a role in the regulation of mineral metabolism, although more research is needed in this area. From above it is evident that the optimum level of IAP is associated with the normal bone functioning and other cellular pathways [1]. The role of IAP in disease conditions and the significance of their inhibitors have led to the discovery of numerous compounds with nitrogen-containing fused heterocyclic skeletons. These compounds have shown promising inhibitory values and many of them have also demonstrated anticancer activity [10]. However, the analysis of the biological data from reported derivatives indicates that there is a lack of selectivity in the mode of inhibition within the same group [11].

The results of previous studies have generated interest in aromatic heterocycles with two nitrogen atoms in their rings. These compounds have been found to exhibit a diverse range of biological activities, including anticancer, analgesic, anti-inflammatory, antimicrobial, antiviral, anticonvulsant, antihistaminic, and anti-HIV properties [12–14]. The significance of hydantoins inhibitor in medicinal chemistry has been emphasized by its recent success [15]. Thiohydantoins are chemical compounds that are similar to hydantoins, but with sulfur atoms replacing one or both of the oxygen atoms in the carbonyl groups, as shown in Fig. 1 [16]. Thiohydantoins and their derivatives have been the subject of extensive research for over 145 years, focusing on their chemistry and properties. The presence of the hydantoin moiety is significant as it serves as a crucial pharmacophore found in numerous biologically active compounds [17].

**Fig. 1:**
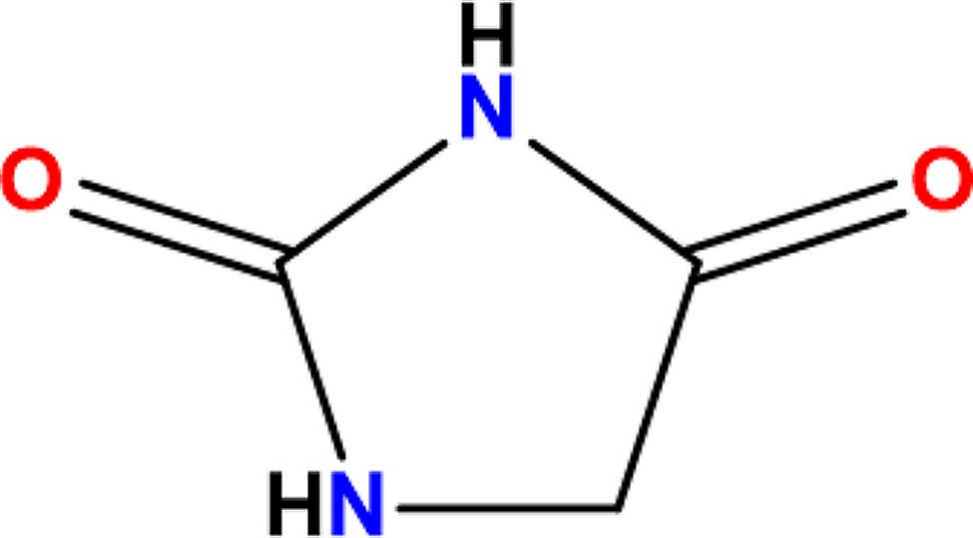
2D structure of 2,4-imidazolidinediones

Thiohydantoins have gained significant attention in the fields of synthetic organic chemistry and biology due to their intriguing chemistry. Compounds containing the 2-thiohydantoin moiety have been extensively studied for their potential applications in various fields, including hypolipidemics [18], anti-carcinogenics [19], anti-mutagenics [20], anti-thyroidals [21], anti-virals [22], anti-microbials [23], anti-ulcer agents [24], anti-inflammatories [25, 26], and herbicides [27]. Enzalutamide is an FDA-approved anti-cancer drug that functions as an androgen receptor antagonist [28]. It contains a 2-thiohydantoin pharmacophore. In addition to the thiohydantoins, the azo derivatives have shown notable activity in various areas such as antimicrobial, antiviral, antidiabetic, anti-melanogenic, anti-ulcer, anticancer, anti-mycobacterial, anti-inflammatory, DNA binding, and chemo-sensing activities [29, 30].

The current study is focused on synthesizing new derivatives of azo-thiohydantoins, taking into consideration the need for IAP inhibitors and the significance of azo and thiohydantoins moieties. The synthesized derivatives were investigated to determine their potential as inhibitors of the targeted enzyme, specifically IAP. The study utilized multiple computational methods, such as Molecular docking, Density functional theory (DFT) calculations, and molecular dynamic simulations, to support the obtained results. These approaches were employed to enhance the understanding of how these derivatives interact with the IAP active pocket. The ADMET profile of all the compounds was calculated in order to evaluate their safety level.

## Methodology

### Material and method

Solvents were pre-treated and distilled as per conventional methods before usage. Reagents were procured from Sigma Aldrich and were used as received, without additional purification. The Stuart SMP3 apparatus was employed to determine the melting point. NMR spectra were taken on a Bruker 300, with 1 H-NMR at 300 MHz and 13 C-NMR at 75.5 MHz. Chemical shifts are presented in ppm, using tetramethylsilane or the solvent’s residual resonance as a reference standard. Reactions’ progress was tracked using thin layer chromatography (TLC). For TLC, we utilized aluminum sheets covered with silica gel F254 from Merck, and compounds were visualized under UV light at 254 and 360 nm wavelengths.

### Synthesis protocol

To a stirred solution of azo-thiosemicarbazone **(6)** (1 mmol) in dry ethanol (20 mL), were added successively the Ethyl chloroacetate (1 mmol) and fused sodium acetate (3 mmol). The reaction mixture was refluxed for 6 h and the progress of the reaction was monitored by TLC. On completion the precipitates formed were cooled and poured into water, filtered and dried. Recrystallized from acetone to afford the purified product which were Azo-Thiohydantoin derivatives (**7**) as given in Scheme 1.

**Fig. 1:**
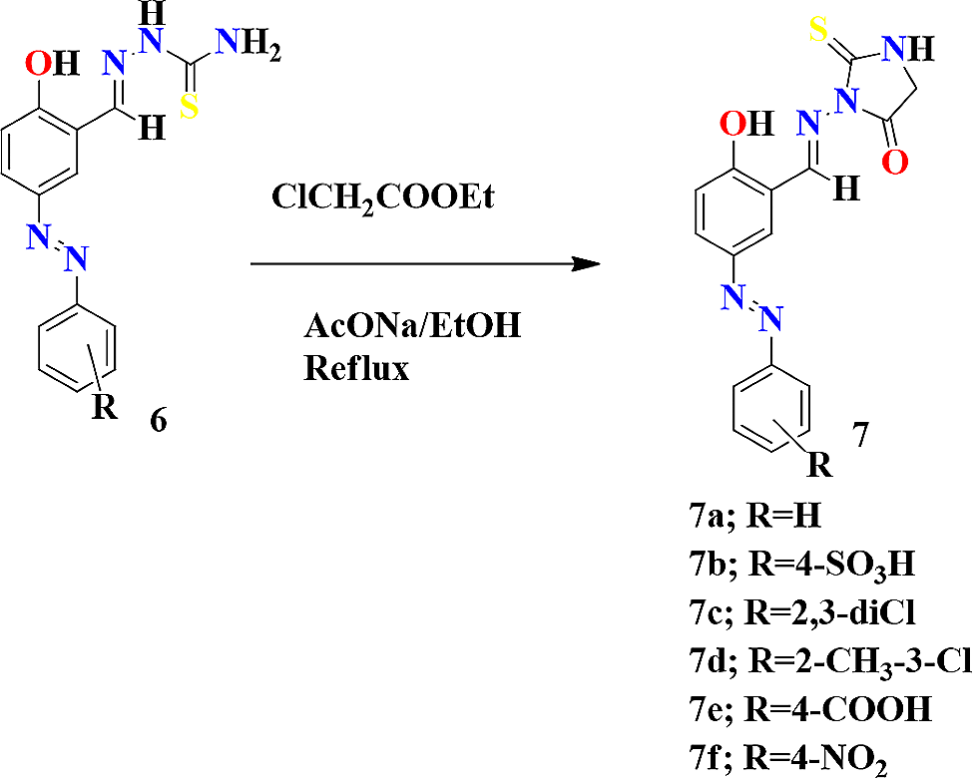
Synthesis of Azo-Thiohydantoin derivatives

### In-vitro studies

#### Alkaline phosphatase inhibition assay

The measurement of intestinal alkaline phosphatase (IAP) activity was conducted using a spectrophotometric method, as described earlier [31, 32]. The assay utilized a reaction mixture consisting of 50 mM Tris-HCl buffer, which included 5 mM MgCl_2_, 0.1 mM ZnCl_2_, and had a pH of 9.5. Additionally, 10 µL of the compound was added to the mixture. The mixture was subjected to pre-incubation with 5 µL of IAP (0.025 U/mL) for a duration of 10 min. The reaction was started by introducing 10 µL of the substrate, which was 0.5 mM p-NPP (para nitrophenyl phosphate disodium salt). Following the addition, the assay underwent an incubation period of 30 min at a temperature of 37 °C. The change in absorbance resulting from the release of p-nitrophenolate was monitored at a wavelength of 405 nm using a Thermo Scientific™ Multiskan GO™ microplate reader. The experiment was repeated three times, with each repetition being done in triplicate. The reference inhibitor for CIAP was L–Phenyl alanine. The calculation of inhibition activity and IC_50_ values were carried out as discussed earlier [33].

### Computational studies

#### Density functional theory

In this study, Density Functional Theory (DFT) was employed using the B3LYP method to accurately determine the electronic density of the molecules. The Gaussian 9w software was utilized to perform comprehensive geometry optimization of the compounds [34]. Additionally, frequency calculations including Frontier Molecular Orbital (FMO) analysis, and global reactivity descriptors were also using the 6-31G basis set [34, 35]. This basis set consists of primitive and Gaussian type orbitals, which are advantageous for accurate FMO analysis. For visualization of the generated files, GaussView 6 software was employed [36].

#### Physicochemical and pharmacokinetic characteristics

The physicochemical and pharmacokinetic attributes of the synthesized derivatives were ascertained through SwissADME (http://www.swissadme.ch/). This computational tool facilitated a comprehensive assessment of several pharmacokinetic parameters, notably absorption, distribution, metabolism, and excretion (ADME) [37]. SwissADME utilize an array of sophisticated algorithms and predictive models to prognosticate both drug-likeness and potential toxicity [38]. The algorithms undergirding the bioavailability radar chart are underpinned by advanced machine learning and statistical methodologies, calibrated against vast molecular datasets with delineated properties. Within the confines of the BOILED-Egg model, salient ADME properties, such as blood-brain barrier (BBB) permeation, passive human gastrointestinal absorption (HIA), and designation as either substrate or non-substrate for permeability glycoprotein, were distinctly identified [39].

#### Molecular Docking Analysis

A molecular docking analysis was conducted to examine the binding interaction between the synthesized derivatives and the targeted protein. The research was conducted by using the homology modeling in which the tissue non-specific ALP was used as the template and model was prepared using MODELLAR as described in our earlier publication. The structure of the model was validated via Ramachandran plot [40]. Prior to conducting docking analysis, the protein structure of IAP was prepared using MGL tools This involved the removal of heteroatoms and water molecules, followed by the addition of polar hydrogen atoms and Kollman charges. Following the rendering of the protein structure, corrections were made to account for any missing residues [41]. The 3D structure of the synthesized derivatives was determined using ChemDraw 3D [42]. Energy minimization was performed to obtain the most stable arrangement of atoms. The dimensions of grid box for active pocket were fixed as follows: (x; 28.093476, y; 19.735517; z; 24.598425) for IAP. Analysis using docking was conducted on the target protein utilizing AutoDock 4.2, with the default genetic algorithm serving as the scoring function. The RMSD value along with re-docking of co-crystal ligand into active pocket was used to validate the docking process.

The newly synthesized derivatives were then docked within the active pocket of target protein, and total 100 different configurations were observed. The most stable configuration with least binding energy was retrieved and analyzed in 2D and 3D forms to elaborate the interactions formed between synthesized compounds and targeted protein. The outcomes of this study are to pave off the way for the designing of novel compounds possessing better binding affinities to the targeted protein.

#### Molecular dynamic simulations

Molecular Dynamic (MD) simulations provide a computational approach for studying the dynamic behavior of molecular interactions within docked complexes [43]. The Nanoscale Molecular Dynamics (NAMD) software version 1.9.3 was utilized in this investigation to make predictions about the dynamic interactions occurring within a protein-ligand complex. The complex with the highest binding energy, as determined through molecular docking results, was selected for the MD simulations. The topology files necessary for the analysis were generated using the CHARM-GUI software [44]. The study employed the CHARMM36 force field parameters to accurately represent the ligand and protein molecules in a system with periodic boundary conditions. In order to replicate the conditions found in the body, the TIP3P water system was chosen as the solvent. Additionally, counter ions (NaCl, 0.15 M) were added to balance the charges in the simulation box. The system underwent equilibration in both the NVT and NPT ensembles using the Berendson thermostat and barostat, respectively. This process occurred over a timestep of 2 fs and at a temperature of 300 K before the main simulation was started. MD simulations were conducted for a duration of 100 nanoseconds. To assess the stability and flexibility of the complex during the simulation, we utilized the Visual Molecular Dynamics (VMD) software version 1.9.3. The analysis involved computing the Root Mean Square Deviation (RMSD) and the Root Mean Square Fluctuation (RMSF) using the Ewald summation method [45].

## Results and discussion

### Chemistry

#### Characterization data of azo-thiohydantoins

##### 7a) 3-((E)-(2-hydroxy-5-((E)-phenyldiazenyl)benzylidene)amino)-2-thioxoimidazolidin-4-one

Light yellow precipitates, Yield = 93%, M.*P* = 236 ^0^C, R_f_ =0.62, (MeOH : CHCl_3_, 2:8), FTIR (cm^− 1^ ) 3605.67 (OH Stretching), 3453.55 (NH Stretching), 3057, 2899 (CH2 str.), 1727 (C = O), 1622.82, 1576.5 (C = N), 1488 (C-H bend CH2), 1346 (C-N str.), 1229.26 (C = S), ^1^H-NMR (300 MHz, DMSO-d6); δ (ppm) 12.126(s, 1H, OH), 11.591(s, 1H, NH), 8.74 (s, 1H, N = CH), 8.22 (dd, 1H, *J* = 1.9, 0.5 Hz), 7.94 (dd, 1H, *J* = 8.3, 1.9 Hz), 7.57 (tt, 1H, *J* = 7.7, 1.3 Hz), 7.45 (dddd, *J* = 8.1, 7.7, 1.5, 0.5 Hz), 7.37–7.50 (4 H, 7.40 (dddd, *J* = 8.1, 1.4, 1.3, 0.5 Hz), 6.89 (dd, 1H, *J* = 8.3, 0.5 Hz), 4.09 (d, 2 H, *J* = 17.7 Hz), ^13^C-NMR (75 MHz, DMSO-d6) : δ(ppm) 176.9, 166.3, 160.5, 152.2, 148.6, 146.5, 129.3, 125.5, 124.7, 122.2, 119.0, 118.0, 116.4, 48.0. Anal. Calcd.for C_16_H_13_N_5_O_2_S : C, 56.63; H, 3.86; N, 20.64; S, 9.45 found: C, 56.61; H, 3.83; N, 20.66; S, 9.43. HRMS Caled for C_16_H_13_N_5_O_2_S + H: 339.0790. Found 339.0787.

##### 7b) 4-((E)-(4-hydroxy-3-((E)-((5-oxo-2-thioxoimidazolidin-1-yl)imino)methyl)phenyl)- diazenyl)benzenesulfonic acid

Brown precipitates, Yield = 89%, M.*P* = 257 0 C, Rf = 0.51, (MeOH : CHCl3, 2:8), FTIR (cm-1 ) 3405 (OH Stretching), 31.85 (NH Stretching), 3027, 2868 (CH2 str.), 1692 (C = O), 1599, 1528, (C = N), 1405 (C-H bend CH2), 1334 (C-N str.), 1168 (C = S), ^1^H-NMR (300 MHZ, DMSO-d6); δ (ppm) 12.126(s, 1H, OH), 11.591(s, 1H, NH), 8.74 (s, 1H, N = CH), 8.60 (dd, 1H, *J* = 1.8, 0.5 Hz), 8.06 (dd, 1H, *J* = 8.1, 1.8 Hz), 7.95 (ddd, 2 H, *J* = 6.4, 1.4, 0.4 Hz), 7.44 (ddd, 2 H, *J* = 6.4, 1.8, 0.5 Hz), 6.98 (dd, 1H, *J* = 8.1, 0.5 Hz), 4.09 (d, 2 H, *J* = 17.7 Hz). ^13^C-NMR (75 MHz, DMSO-d6): δ(ppm) 176.9, 166.3, 160.5, 153.2, 148.6, 146.1, 138.1, 127.5, 125.5, 124.7, 121.9, 118.0, 116.4, 48.1. Anal. Calcd.for C_16_H_13_N_5_O_5_S_2_ : C, 45.82; H, 3.12; N, 16.70; S, 15.29 found: C, 45.84; H, 3.10; N, 16.72; S, 15.27. HRMS Caled for C_16_H_13_N_5_O_5_S_2_ + H: 419.0358. Found 419.0354.

##### 7c)3-((E)-(5-((E)-(2,3-dichlorophenyl)diazenyl)-2-hydroxybenzylidene)amino)-2 thioxoimidazolidin-4-one

Orange precipitates, Yield = 95%, M.*P* = 249 ^0^C, R_f_ =0.54, (MeOH : CHCl_3_, 2:8), FTIR (cm^− 1^ ) 3609.27 (OH Stretching), 3459.55 (NH Stretching), 3057, 2896 (CH2 str.), 1734 (C = O), 1620.79, 1575.5 (C = N), 1487 (C-H bend CH2), 1345 (C-N str.), 1228.26 (C = S), 766 (C-Cl str. arom.), ^1^H-NMR (300 MHZ, DMSO-d6); δ (ppm) 12.26(s, 1H, OH), 11.691(s, 1H, NH), 4.09 (d, 2 H, *J* = 17.7 Hz), 6.88 (dd, 1H, *J* = 8.4, 0.5 Hz), 6.96 (dd, 1H, *J* = 6.7, 1.1 Hz), 7.22 (dd, 1H, *J* = 7.8, 6.7 Hz), 7.62 (dd, *J* = 8.4, 1.9 Hz), 7.64 (dd, *J* = 7.8, 1.1 Hz), 8.18 (dd, 1H, *J* = 1.9, 0.5 Hz), 8.70 (s, 1H). ^13^C-NMR (75 MHz, DMSO-d6): δ(ppm) 176.9, 166.3, 160.5, 152.4, 148.6, 146.1, 133.7, 130.1, 127.5, 125.5, 123.1, 119.0, 118.0, 117.3, 116.4, 48.0. Anal. Calcd.for C_16_H_11_Cl2N_5_O_2_S : C, 47.07; H, 2.72; N, 17.15; S, 7.85 found: C, 47.05; H, 2.76; N, 17.12; S, 7.83. HRMS Caled for C_16_H_11_Cl2N_5_O_2_S + H: 407.0011. Found 407.0008.

##### 7d) 3-((E)-(5-((E)-(3-chloro-2-methylphenyl)diazenyl)-2-hydroxybenzylidene)amino)-2-thioxoimidazolidin-4-one

Yellow precipitates, Yield = 89%, M.*P* = 296 ^0^C, R_f_ =0.51, (MeOH : CHCl_3_, 2:8), FTIR (cm^− 1^ ) 3612.67 (OH Stretching), 3450.68 (NH Stretching), 3059, 2899 (CH2 str.), 1726 (C = O), 1622.82, 1575.5 (C = N), 1476(C-H bend CH2), 1345 (C-N str.), 1228.26 (C = S), 764 ,782 (C-Cl str. arom.), ^1^H-NMR (300 MHZ, DMSO-d6); δ (ppm) 12.36(s, 1H, OH), 11.631(s, 1H, NH), 8.72 (1H, s), 8.21 (1H, dd, *J* = 1.9, 0.5 Hz), 7.94 (1H, dd, *J* = 8.3, 1.9 Hz), 7.41 (1H, dd, *J* = 7.9, 1.2 Hz), 7.16 (1H, dd, *J* = 8.1, 7.9 Hz), 6.85 (dd, *J* = 8.3, 0.5 Hz), 6.83–6.89 (2 H, 6.86 (dd, *J* = 8.1, 1.2 Hz), 4.09 (2 H, d, *J* = 17.7 Hz), 2.25 (s, 3 H), ^13^C-NMR (75 MHz, DMSO-d6) : δ(ppm) 176.9,166.3, 160.5, 148.6, 147.4,146.1, 134.4, 130.1, 126.7, 125.5, 124.8, 120.8, 119.0, 118.0, 116.4, 48.0, 13.9. Anal. Calcd.for C_17_H_14_ClN_5_O_2_S: C, 52.65; H, 3.64; N, 18.06; S, 8.27 found: C, 52.63; H, 3.62; N, 18.04; S, 8.25 HRMS Caled for C_17_H_14_ClN_5_O_2_S + H: 387.0557. Found 387.0553.

##### 7e) 4-((E)-(4-hydroxy-3-((E)-((5-oxo-2-thioxoimidazolidin-1-yl)imino)methyl)phenyl) diazenyl)benzoic acid

Light yellow precipitates, Yield = 88%, M.*P* = 283 0 C, Rf = 0.60, (MeOH : CHCl3, 2:8), FTIR (cm-1 ) 3474.58 (OH Stretching), 3434.58 (NH Stretching), 3035 (Ar-H), 2924 (CH2 str.), 1719 (C = O), 1610, 1535 (C = N), 1435 (C-H bend CH2), 1317 (C-N str.), 1145 (C = S), 1 H-NMR (300 MHZ, DMSO-d6); δ (ppm) 12.508 (s, 1 H, OH COOH), 11.507(s, 1 H, NH), 10.757 (s, 1 H, OH), 8.696 (dd, 1 H, J = 1.9, 0.5 Hz), ), 8.30 (ddd, 1 H, J = 8.3, 1.5, 0.5 Hz), 8.013 (d, 2 H, J = 8.3, Hz), 7.89 (ddd, 1 H, J = 8.3, 1.9 Hz) 7.86 (s, 1 H CH = N-). 7.77 (d, 2 H, J = 8.3), 4.72 (s, 2 H, Thiohydantoin), 13 C-NMR (75 MHz, DMSO-d6): δ(ppm) 181.5 (C = S), 177.2(C = O), 167.5(COOH), 162.2, 158.8, 151.8, 137.9, 134.3, 131.4, 123.4, 121.7, 118.5, 116.7, 112.1, 63.3. Anal. Calcd.for C17H13N5O4S : C, 53.26; H, 3.42; N, 18.27; S, 8.36 found: C, 53.24; H, 3.40; N, 18.25; S, 8.34 HRMS Caled for C17H13N5O4S + H: 383.0688. Found 383.0685.

##### 7f) 3-((E)-(2-hydroxy-5-((E)-(4-nitrophenyl)diazenyl)benzylidene)amino)-2-thioxoimidazolidin-4-one

Redish brown precipitates, Yield = 86%, M.*P* = 271–273 ^0^C, R_f_ =0.61, (MeOH: CHCl_3_, 2:8), FTIR (cm-1 ) 3303 (OH Stretching), 3193 (NH Stretching), 3031, 2848 (CH2 str.), 1696 (C = O), 1598, 1531 (C = N), 1480 (C-H bend CH2), 1327 (C-N str.), 1256 (C = S), ^1^H-NMR (300 MHZ, DMSO-d6); δ (ppm) 12.12(s, 1H, OH), 11.581(s, 1H, NH), 8.70 (s, 1H), 8.58 (dd, 1H, *J* = 1.9, 0.5 Hz), 8.34 (ddd, 2 H, *J* = 8.7, 1.8, 0.5 Hz), 8.03 (dd, 1H, *J* = 8.3, 1.9 Hz), 7.41 (ddd, 2 H, *J* = 8.7, 1.8, 0.5 Hz), 6.96 (dd, 1H, *J* = 8.3, 0.5 Hz), 4.09 (d, 2 H, *J* = 17.7 Hz). ^13^C-NMR (75 MHz, DMSO-d6): δ(ppm) 176.9, 166.3, 160.5, 148.6, 146.1, 145.7, 140.5, 125.5, 125.2, 119.1, 118.0, 117.0, 116.4, 48.0. Anal. Calcd.for C_16_H_12_N_6_O_4_S : C, 50.00; H, 3.15; N, 21.86; S, 8.34 found: C, 50.02; H, 3.17; N, 21.84; S, 8.32 HRMS Caled for C_16_H_12_N_6_O_4_S + H: 384.0641. Found 384.0639.

Characterization spectra i.e. FTIR, ^1^H-NMR and ^13^C-NMR data are given in Figures S1-S3 (supplementary file).

#### Alkaline phosphatase inhibition assay

The derivative **7e**, and **7f** showed comparable enzyme inhibition activity i.e. 0.308 ± 0.065, and 0.836 ± 0.097 µM, respectively. The structure activity relationship of these derivatives displayed the combine effect of both mesomeric and inductive effect of various substituted groups i.e., carbonyl and Nitro group on parent ring structure. Carbonyl and nitro group functional groups are strong electron withdrawing groups due to greater electronegativity and impart specific physicochemical properties to synthesized derivatives leading to enhanced anti-enzymatic potential.

The **7e** derivatives exhibited greater inhibitory activity as compared to the standard inhibitor i.e., L–Phenyl alanine IC_50_ = 80.2 ± 1.1 µM. The substitutional effect on parent molecule was also confirmed in case of **7a**, **7b**, **7c**, and **7d** derivative with further reduction of enzyme inhibitory activity. In case of **7a** compound the phenyl ring without any other substitution showed weak mesomeric effect that resulted in least inhibitory effect i.e. 4.847 ± 0.985. IC_50_ values of synthesized derivatives **7a-7f** are given in Table 1.

**Table 1:** Intestinal Alkaline phosphatase inhibitory activity of **7a-7f** derivatives

Compound	Alkaline phosphatase IC_50_ ± SEM (µM)	Compound	Alkaline phosphatase IC_50_ ± SEM (µM)
**7a**	4.847 ± 0.985	**7d**	3.149 ± 0.853
**7b**	1.276 ± 0.214	**7e**	0.308 ± 0.065
**7c**	3.071 ± 0.687	**7f**	0.836 ± 0.097
L–Phenyl alanine	80.2 ± 1.1		

Values are presented as Mean ± SEM (Standard error of the mean)

#### Density functional theory calculations

The study focused on conducting a thorough analysis of the energies of the Highest Occupied Molecular Orbital (HOMO) and Lowest Unoccupied Molecular Orbital (LUMO) using Density Functional Theory (DFT) calculations. The DFT calculations utilized the B3LYP method along with widely recognized 631-G basis set, renowned for its ability to accurately depict molecular properties. Table 2 provides the HOMO-LUMO energies, which are crucial for comprehending the electronic structure and reactivity of the compounds being studied. Table 2 presents a concise overview of the significant findings derived from our study, which specifically examined the electronic characteristics of the compounds being analyzed.

**Table 2:** Optimization energies, HOMO and LUMO energies, and their gap estimated in gas

Codes	Optimization Energy	Dipole moment	Polarizability (α)	HOMO (eV)	LUMO (eV)	HOMO-LUMO ($\varDelta$eV)
**7a**	-1439.52	1.81	274.29	-0.21	-0.08	0.13
**7b**	-2063.07	6.03	325.85	-0.22	-0.11	0.11
**7c**	-2358.65	4.43	296.90	-0.22	-0.09	0.12
**7d**	-1938.40	3.01	300.32	-0.21	-0.09	0.12
**7e**	-1628.01	6.40	305.49	-0.22	-0.11	0.11
**7f**	-1643.94	6.36	312.42	-0.22	-0.12	0.10

**Fig. 2:**
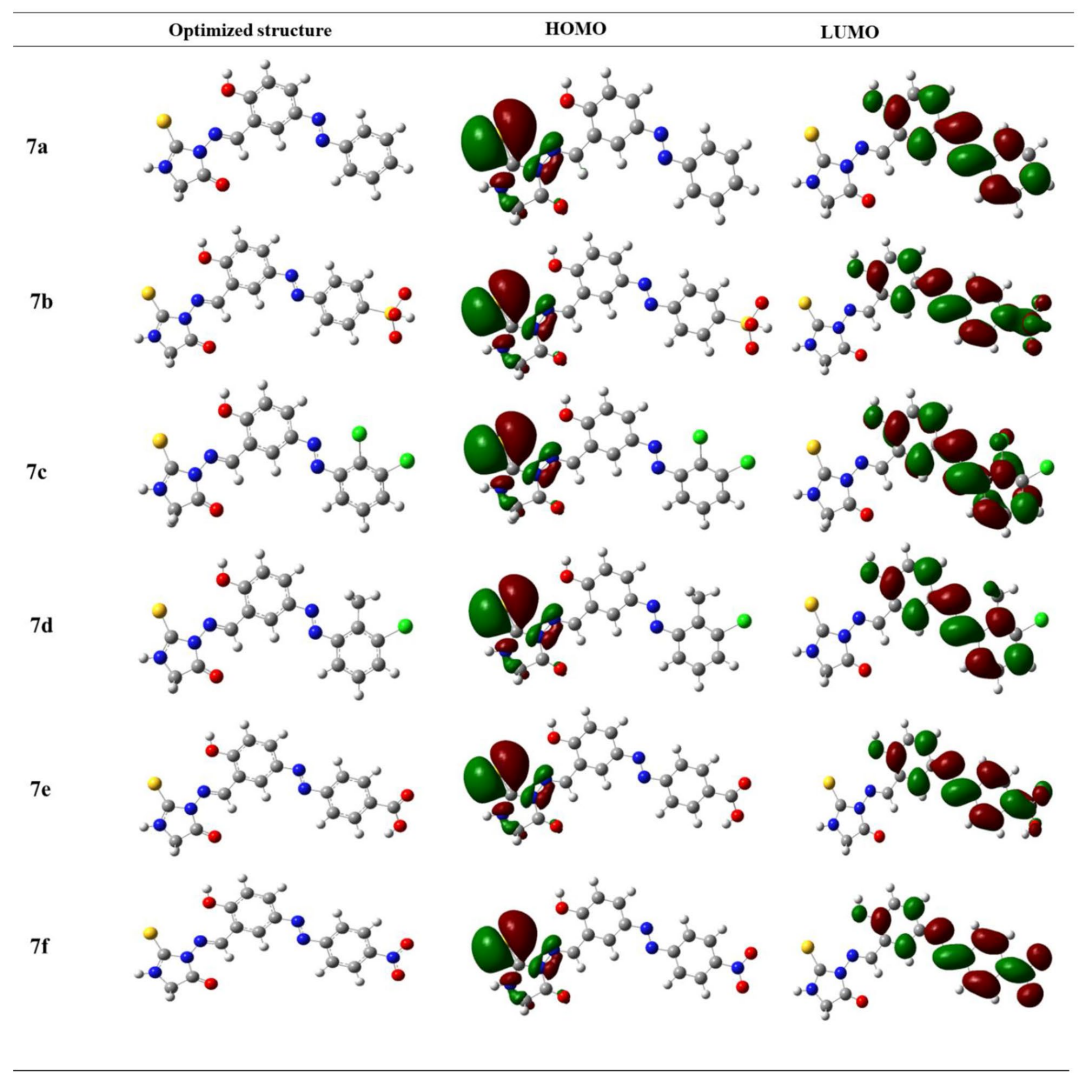
HOMO and LUMO orbitals, along with the optimized molecular structure, for the studied compound using DFT/B3LYP/6-31G’ calculations

The Table 2 contains the optimization energies, as well as the energies of the Highest Occupied Molecular Orbital (HOMO) and Lowest Unoccupied Molecular Orbital (LUMO). Moreover, it provides the computed difference between the highest occupied molecular orbital (HOMO) and the lowest unoccupied molecular orbital (LUMO) energies, which is a vital factor in comprehending the electronic characteristics and reactivity of the molecules, especially in the gaseous state. The optimized structures and highest occupied molecular orbital (HOMO) and lowest unoccupied molecular orbital (LUMO) orbitals are depicted in Fig. 2.

A molecule with a larger HOMO-LUMO energy gap is considered to be “hard.” This means that it possesses higher kinetic stability and is less prone to undergo chemical reactions. The larger gap indicates that there is a greater energy barrier between the occupied and unoccupied orbitals, which hinders electron transfer and reactivity. Additionally, a “hard” molecule tends to have lower polarization, as the electron density is more evenly distributed. On the other hand, a molecule with a smaller HOMO-LUMO energy gap is referred to as “soft.” Such molecules exhibit lower stability and are more reactive. The smaller gap indicates a lower energy barrier, allowing for easier electron transfer and facilitating chemical reactions. These molecules tend to be more polarized, with uneven distribution of electron density.

By analyzing the HOMO-LUMO energy band gap, we can gain insights into the stability, reactivity, and polarization tendencies of a molecule, which are important factors in understanding its chemical behavior and interactions with other substances.

#### Global chemical reactivity descriptors

By using HOMO LUMO energy values, we evaluated the following parameters by using their respective formulas: Hardness: η = 1/2(ELUMO - EHOMO); Hardness essentially measures the resistance of a chemical system to change. In simpler terms, a molecule’s ‘hardness’ indicates its reactivity and stability. A larger gap between HOMO and LUMO energies means greater stability and lower reactivity. Softness: S = 1/2η; Softness indicates how easily a molecule can donate or accept electrons, essentially a measure of its flexibility or adaptability in reactions. Electronegativity: χ = -1/2(ELUMO + EHOMO); Electronegativity represents the tendency of a molecule to attract electrons. Chemical potential: µ = - χ; This represents the potential energy change of a system when an additional electron is added. Electrophilicity index: ω = µ/2η gauges a molecule’s likelihood to accept electrons. Higher values suggest the molecule is a good electron acceptor, making it more electrophilic. Table 3 provides an overview of the Global Reactivity Descriptors for compounds **7a-7f**. These descriptors offer valuable information about the reactivity and stability of each compound.

**Table 3:** An overview of the Global Reactivity Descriptors for synthesized compounds (**7a-7f**)

Codes	Chemical Potential µ (eV)	Electronegativity X (eV)	Hardness ƞ (eV)	softness S (eV-1)	Electrophilicity index ω (eV)
**7a**	-0.148	0.148	0.064	7.779	0.169
**7b**	-0.165	0.165	0.054	9.328	0.255
**7c**	-0.155	0.155	0.061	8.254	0.198
**7d**	-0.152	0.152	0.062	8.009	0.184
**7e**	-0.161	0.161	0.056	8.906	0.232
**7f**	-0.170	0.170	0.049	10.172	0.293

In particular, compound **7f** and **7e** stands out with an Electronegativity value of -0.170 eV and − 0.161 eV respectively. This value signifies its strong electron-attracting capability, indicating that it has a high power to draw electrons towards itself. Consequently, compound **7f** is classified as the “softest” compound among the studied compounds. This means that it exhibits lower stability, higher reactivity, and a higher tendency to undergo chemical reactions. Furthermore, compound **7f** also demonstrates a high electrophilicity index, which measures the molecule’s ability to accept electrons through its chemical potential. This index indicates that compound **7f** has a pronounced capability to accept electrons, making it an attractive candidate for electron-transfer reactions.

In summary, the analysis of Global Reactivity Descriptors in Table 3 reveals that compound **7f** exhibits the characteristics of a “soft” molecule, with high electrophilicity and a strong ability to attract electrons. These findings provide valuable insights into the reactivity and chemical behavior of compound **7f** compared to the other compounds studied.

### Physicochemical and pharmacokinetic characteristics

**Fig. 3:**
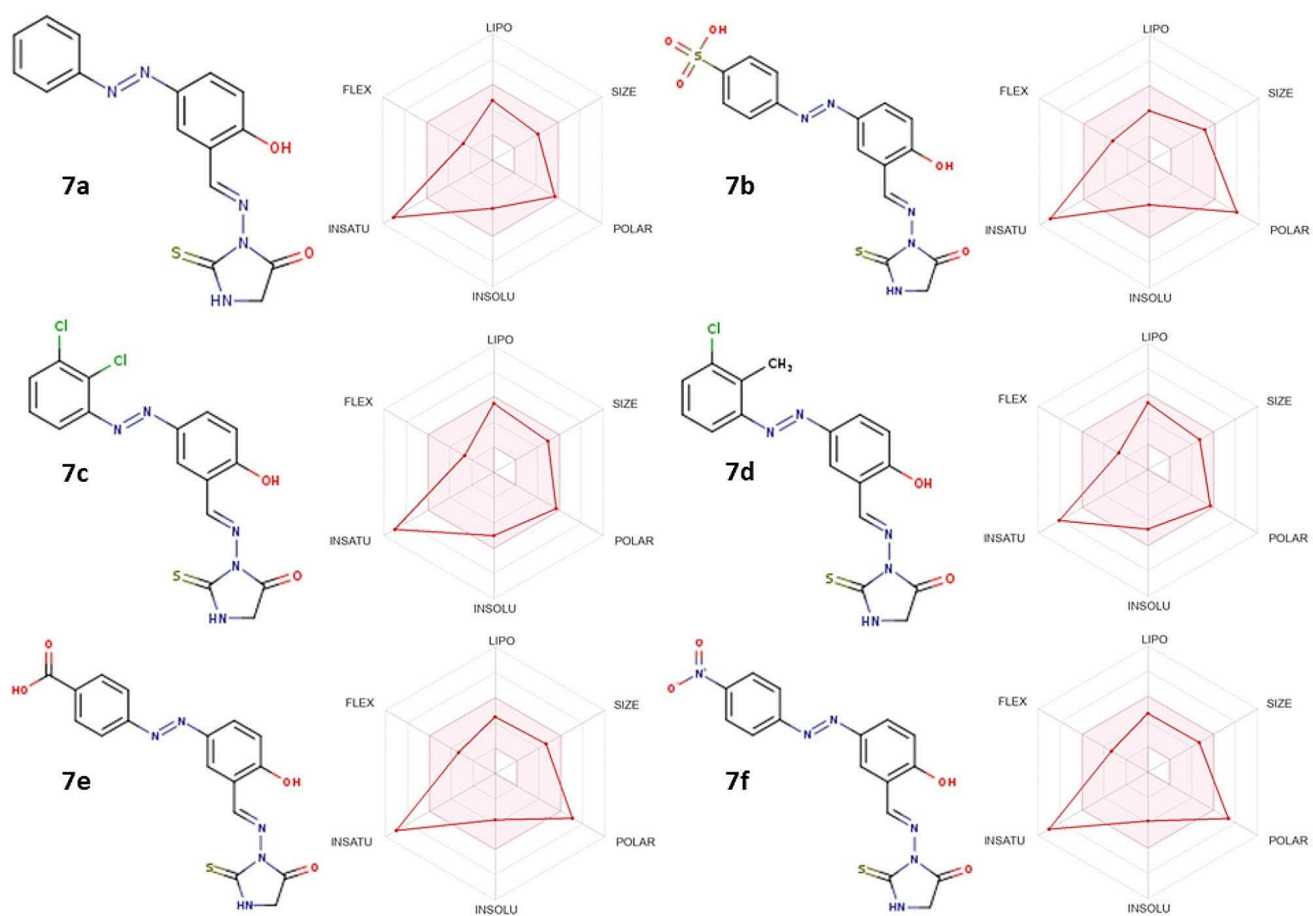
Structures of synthesized derivatives and radar charts describing their physicochemical and pharmacokinetic properties

The synthesized derivatives (**7a-7f**) underwent evaluation for their physicochemical properties using the SwissADME tool. Among these, the derivatives **7a**, **7c**, and **7d** demonstrated high oral bioavailability. The comprehensive physicochemical and pharmacokinetic profiles of these derivatives are described in Fig. 3 and supplementary file (Table S1).

In terms of solubility, all derivatives displayed effective dissolution. However, **7a**, **7b**, **7e**, and **7f** emerged as the most soluble. The compounds **7a**,**7c** and **7d** displayed a more pronounced drug likeness characteristic in comparison to all synthesized compounds. Derivatives **7a**, **7c**, and **7d** exhibited high passive human gastrointestinal absorption, while **7b**, **7e**, and **7f** showed low levels.

**Fig. 4:**
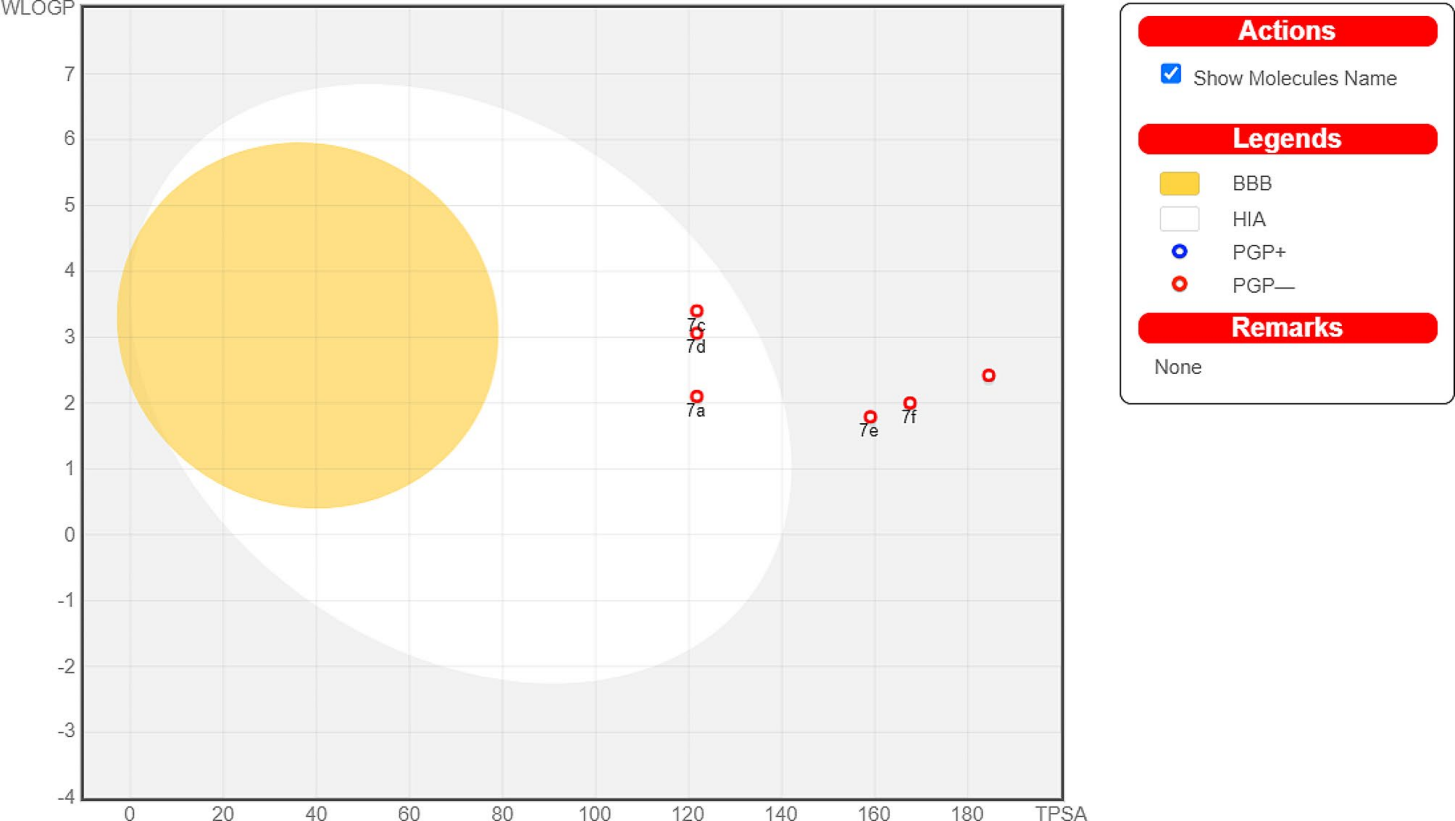
Boiled egg presentation of synthesized derivatives (**7a-7f**)

The graphical representation, illustrated by the boiled egg diagram as shown in Fig. 4, aids in the visualization of drug likeness properties, especially in relation to the blood-brain barrier (BBB) permeation. Within this diagram the yolk designated as compounds with a potential to permeate the BBB. Notably, none of the derivatives permeated the blood-brain barrier (BBB). While the white region denoted as compounds unlikely to cross the BBB and are not P-glycoprotein (P-gp) substrates. The analysis showed that the derivatives **7b**, **7e**, and **7f** were identified as P-gp substrates. The external region of the egg identified as the compounds anticipated to be P-gp substrates, implying they might exhibit reduced absorption and distribution due to potential active efflux from cells.

**Fig. 5:**
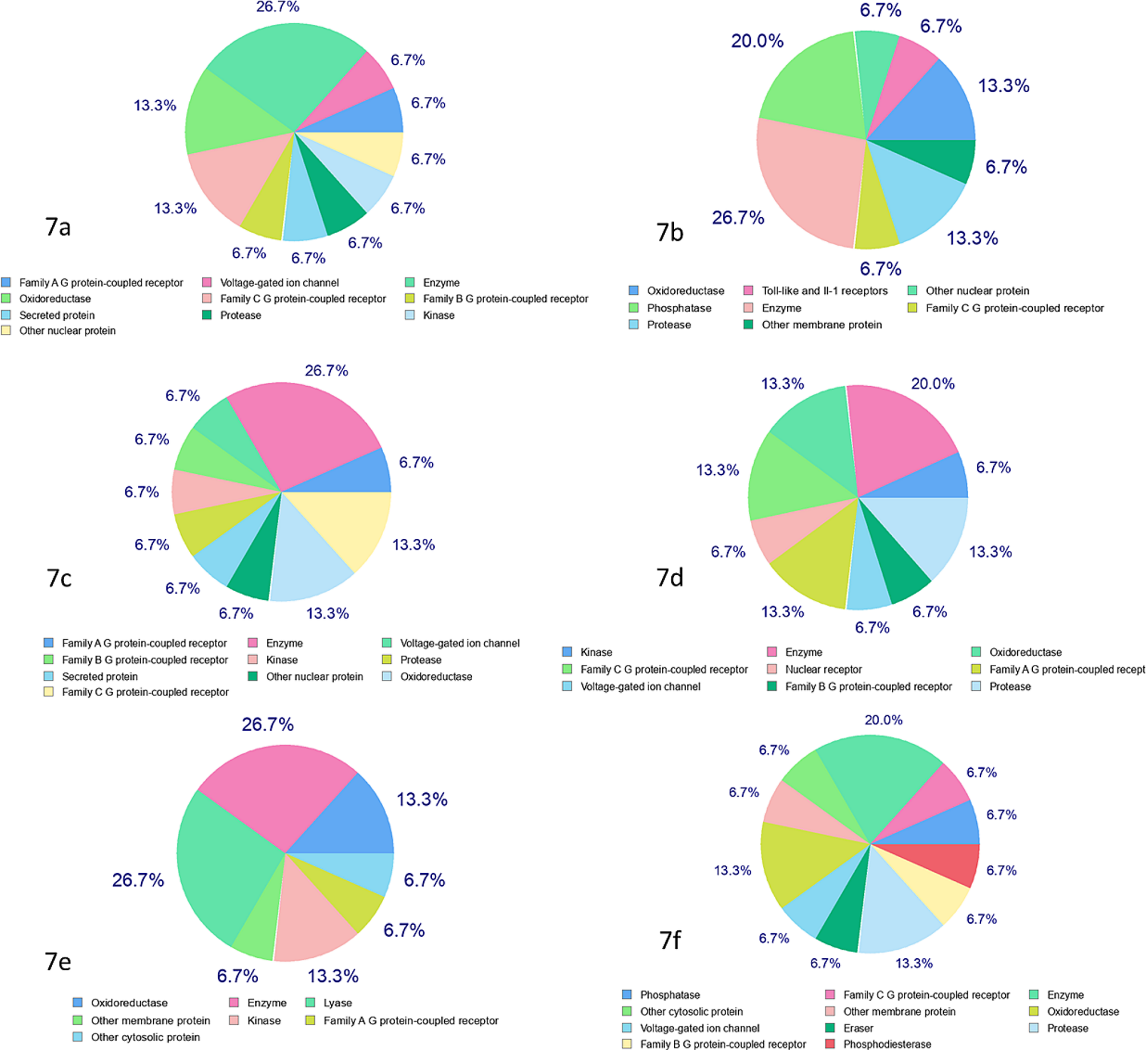
Predicted classes of proteins using Swiss ADME’s target prediction tool

In the boiled egg diagram, the x-axis conventionally illustrates the topological polar surface area (TPSA), while the y-axis depicts the Wildman-Crippen LogP (WLOGP), serving as a metric for lipophilicity based on molecular structure. While the boiled egg diagram offers a streamlined visualization of the intricate factors influencing ADME, it remains invaluable for preliminary evaluations in drug discovery endeavors. Additionally, SwissTarget Prediction tool was utilized to predict the potential protein targets ranked by the probability score as shown in Fig. 5. The higher the score, the more likely the molecule binds to that protein target. However, the results were further validated with experimental data for a conclusive understanding.

#### Molecular docking analysis

Molecular docking is an important computational tool for determination of interactions between ligand and protein at molecular level. In current study we were aimed to predict the binding scores of synthesized derivatives with homology model of targeted protein, namely intestinal alkaline phosphatase (IAP). Homology models was retrieved from our previously reported study [1].

In recent docking experiments, the interaction of compounds **7f** and **7e** with the active site of IAP demonstrated notable binding affinities, with binding energies of -7.8 kcal/mol and − 7.9 kcal/mol respectively as given in Table 4. Upon in-silico evaluation, it was noted that compound **7f** possessed the most favorable binding energy, whereas the **7e** derivative showcased the second highest. Despite the subtle structural variations between the derivatives **7e** and **7f**, there is a pronounced disparity in their binding energies, which is further elaborated by in-vitro analyses.

**Table 4:** Molecular docking scores of compounds with Intestinal Alkaline Phosphatase (IAP)

Protein	Ligand Codes	Binding energy Kcal/mol	Hydrogen bond forming residues	Hydrophobic interactions residues
Intestinal Alkaline phosphatase (homology model)	**7a**	-7.3	SER363	VAL361, TYR393, LEU392, TYR399, ARG406
	**7b**	-6.9	ARG87, HIS153	HIS432
	**7c**	-7.2	THR345	ARG73, GLU339, ARG442, ALA70
	**7d**	-7.4	SER363	TYR399, LEU392, ARG406, PHE401
	**7e**	-7.9	ARG314, GLU321, TYR276, GLN108	ARG166, HIS432, ZN530, HIS317, HIS320, ASP316
	**7f**	-7.8	SER92, HIS432, HIS317	THR431, MG532, ARG166, ASP316, ZN530, PHE107
	**L–Phenyl alanine**	-5.4	No	LEU329, ALA424, GLN422, TYR399, ARG406

**Fig. 6:**
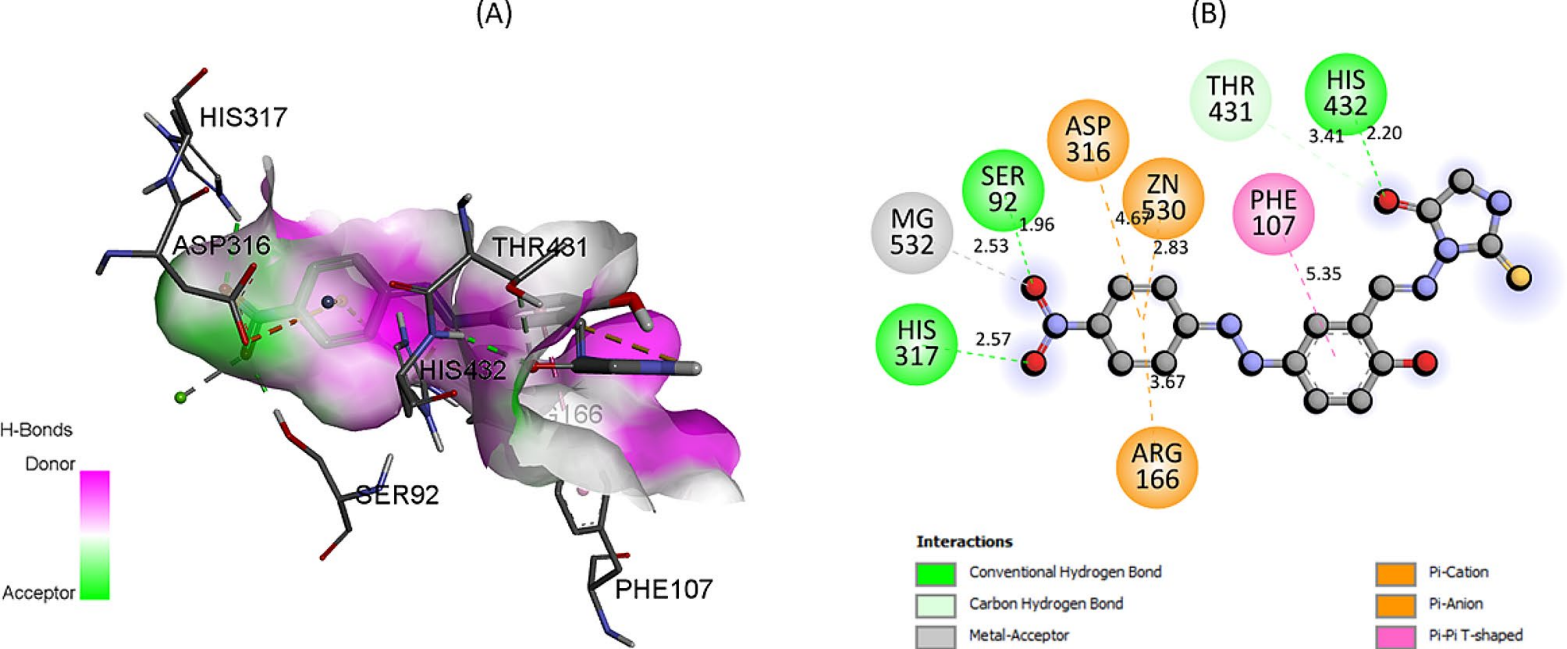
The predicted 2D and 3D binding pose of compound 7f within the active pocket of IAP

The two-dimensional and three-dimensional binding mode of **7f** derivative with IAP is shown in Fig. 6. Analysis revealed that the amino acid residues SER92, HIS432, and HIS317 engage in conventional hydrogen bonding with the **7f** derivative. Concurrently, the THR431 residue establishes a carbon-hydrogen bond with the **7f.** It’s imperative to highlight that the metal ions, specifically Mg532 and Zn530, play a pivotal role in this binding interaction. While Mg532 functions as a metal acceptor, Zn530 contributes through a pi-cation interaction. This intricate binding interactions attributed the potential significance and specificity of these interactions in the context of IAP’s active site.

**Fig. 7:**
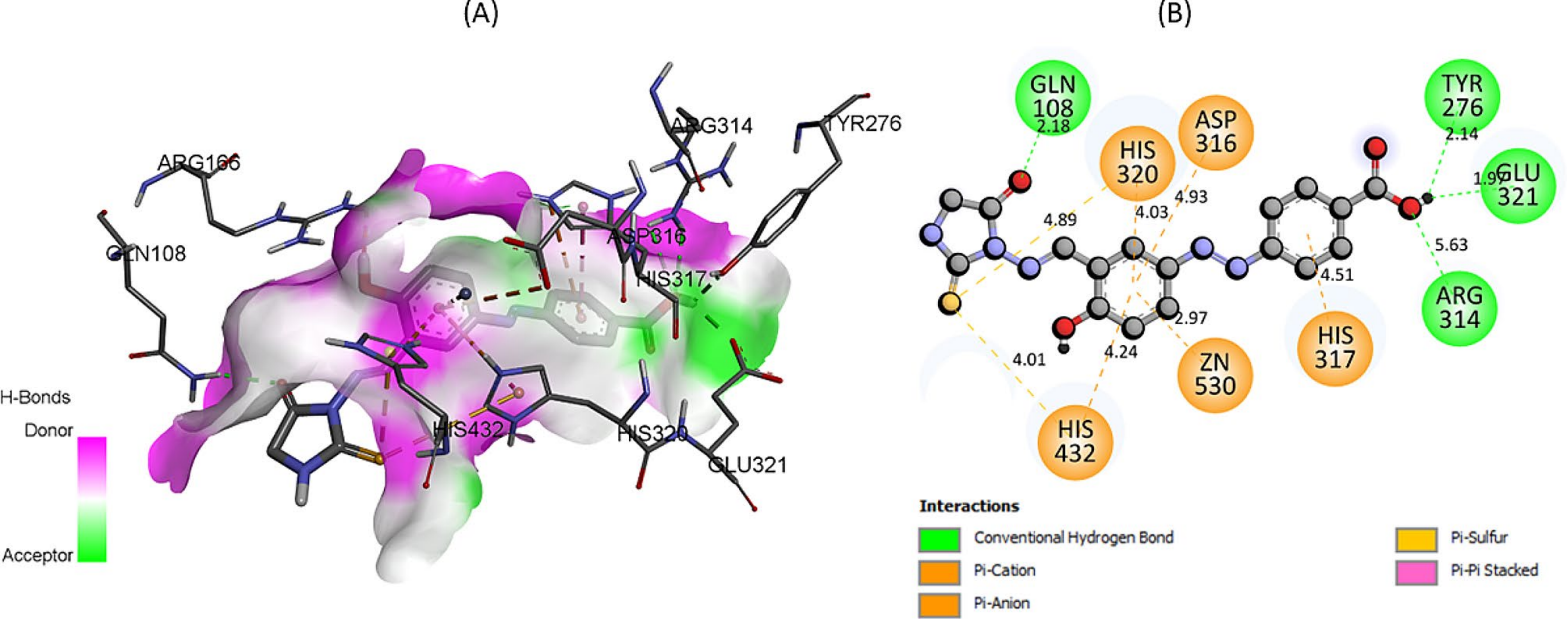
The predicted 2D and 3D binding pose of compound **7e** within the active pocket of IAP

In Fig. 7, a detailed representation of both the two-dimensional and three-dimensional binding modes of the **7e** derivative with IAP is presented. Upon rigorous analysis, it becomes evident that certain amino acid residues, namely ARG314, GLU321, TYR276, and GLN108, engage in conventional hydrogen bonding interactions with compound 7e. This underlines the pivotal roles these specific residues might play in facilitating the binding and stabilization of the 7e derivative within IAP’s active site.

Moreover, other residues such as HIS432, HIS317, HIS320, and ASP316, along with the metal ion Zn530, are observed to participate in pi-anion interactions. These interactions are characterized by the overlap between the pi-system of an aromatic residue and an anionic moiety, which results in a stabilizing force. Such interactions emphasize the intricate nature of the binding, where multiple residues and forces cooperate to accommodate the ligand, in this case, the 7e derivative, within the protein’s binding pocket. 2D and 3D binding modes of **7a-7d** are given in supplementary file (Figure S4-S7). These results were further validated through *in-vitro* analysis as described next.

**Fig. 8:**
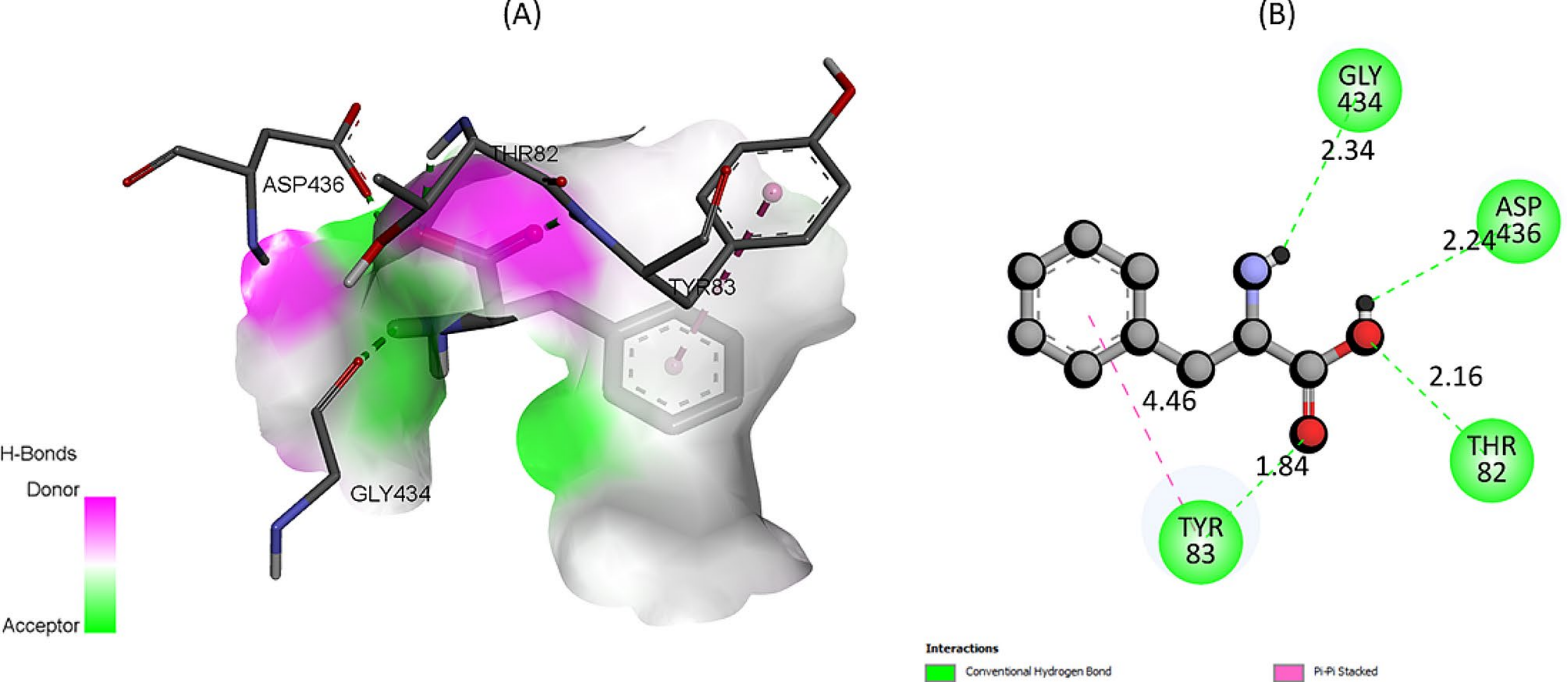
The predicted 2D and 3D binding pose of compound L–Phenyl alanine within the active pocket of IAP

Figure 8 provides a comprehensive depiction of the binding modes between L–Phenyl alanine and IAP, showcasing both two-dimensional and three-dimensional perspectives. Through thorough examination, it is apparent that specific amino acid residues—namely GLY434, ASP436, THR82 and TYR83—establish the conventional hydrogen bond with the L-phenylalnine. Additionally, TYR83 participate in pi-pi stacked interaction with the standard inhibitor compound, L–Phenyl alanine.

#### Molecular dynamics simulations

Molecular Dynamics (MD) simulations were executed to investigate the conformational dynamics of both the apo protein and its complex with the ligand. The stability of these structures was quantitatively examined using Root Mean Square Deviation (RMSD) and Root Mean Square Fluctuation (RMSF) over the simulation duration.

**Fig. 9:**
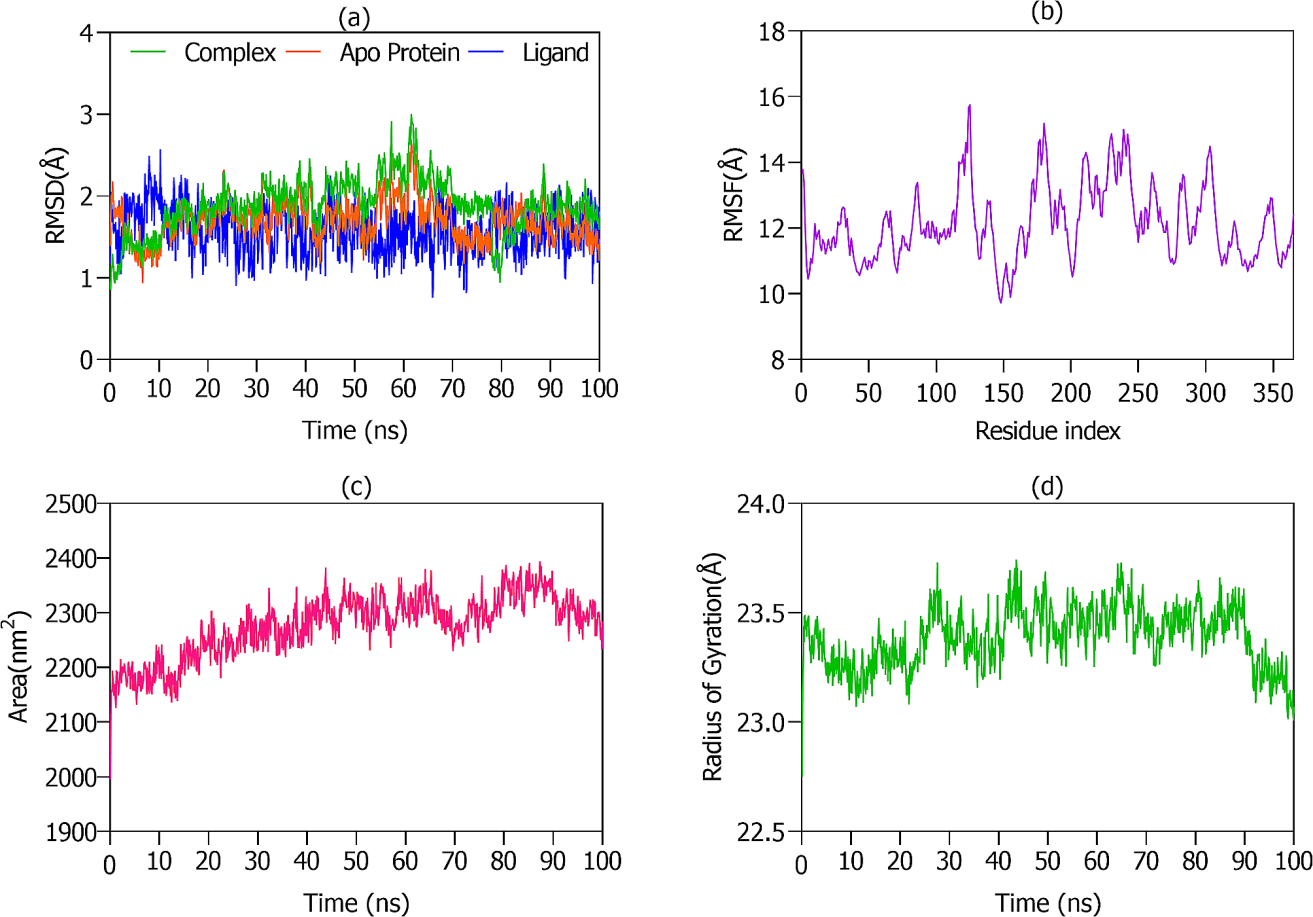
(**a**) RMSD plots illustrating the dynamics of ligand **7e**, IAP protein, and their combined complex. (**b**) RMSF graph highlighting the fluctuation and flexibility within the IAP protein residues. (**c**) Time-course SASA plot depicting solvent exposure regions of the IAP protein. (**d**) Temporal trajectory of the IAP protein’s Radius of Gyration (Rg), indicating its conformational compactness

From the RMSD plot (Fig. 9 (a)), it is discernible that the protein-ligand complex offers superior stability in comparison to the isolated apo protein and ligand. Specifically, Fig. 9(a) elucidates that the complex retains consistent conformational steadiness throughout the simulation, as evinced by an RMSD of 1.88Å. Intriguingly, the apo protein and ligand **(7e)** independently showcased robust stability, evidenced by RMSD values of 1.70Å and 1.60Å, respectively.

RMSF calculations, depicted in Fig. 9(b), reveal an average fluctuation of 12.14Å, emphasizing the dynamic flexibility of the amino acid residues, a feature pivotal for potent protein-ligand interactions. Notably, the ligand (**7e**) remained anchored within the protein’s active site during the simulation. The pronounced peaks in Fig. 9(b) signify residues undergoing significant vibrational oscillations, while the troughs pinpoint residues fostering robust hydrophobic and hydrophilic interactions with the ligand.

The Solvent Accessible Surface Area (SASA) plot (Fig. 9(c)) underscores segments of the protein exhibiting heightened solvent exposure, suggesting these regions possess inherent flexibility and are less compact. Such domains potentially partake in the dynamic functional activity and molecular interplay of the protein. Conversely, the troughs in the SASA plot allude to internally situated residues, integral to the molecule’s structural stability and folding.

Radius of Gyration (Rg) analysis, represented in Fig. 9(d), provides a lens into the compactness of the protein’s conformation. Periods with lower Rg values correlate with instances where the protein assumes a more condensed, globular structure, indicative of its stabilized states. By juxtaposing insights from both SASA and Rg plots, one can derive a comprehensive understanding of the evolving conformation and resilience of the apo protein across various conditions. Together, these analytical tools offer invaluable perspectives into the nuanced structure-function dynamics of biomolecules, facilitating advanced studies in protein stability and mechanics.

**Fig. 10:**
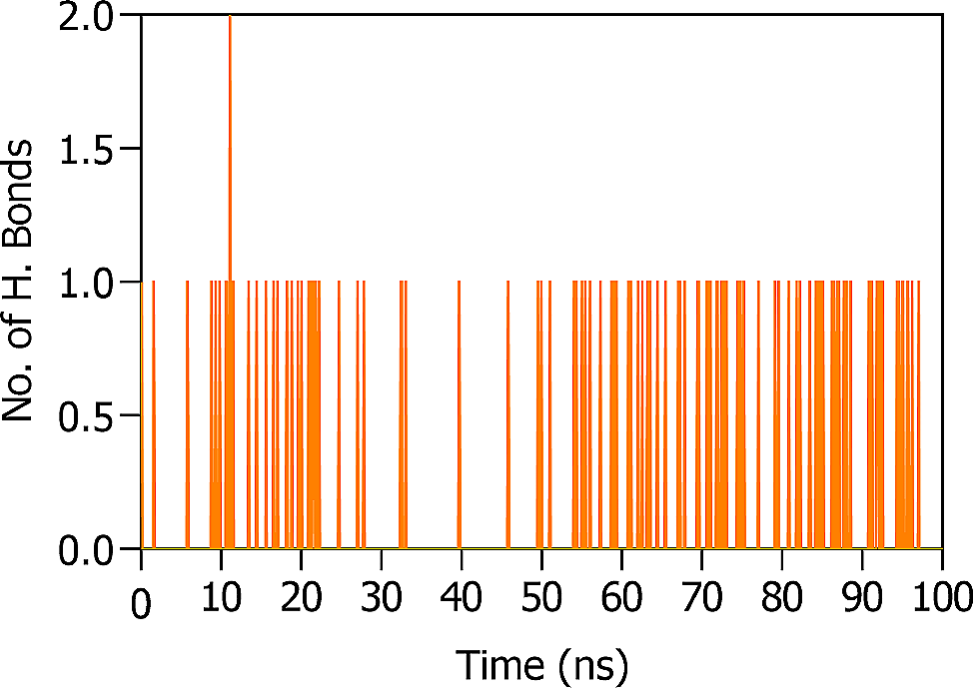
Hydrogen bond analysis of 7e with IAP during MD simulations

Electrostatic interactions between ligand **7e** and the IAP protein are intricately shaped by the amino acid residues responsible for hydrogen bond formation. An in-depth analysis of Table 5; Fig. 10 highlights the pivotal roles of specific IAP residues in fostering a strong hydrogen bond network with the ligand.

**Table 5:** Hydrogen Bond Donor and Acceptor Groups of 7e and IAP protein

Sr #	Hydrogen bond Donor	Hydrogen bond Acceptor	Occupancy
1	7e-Side	GLU321-Side	0.18%
2	7e -Side	HSD432-Side	0.09%
3	SER429-Side	7e -Side	1.25%
4	7e -Side	HSD360-Side	7.86%
5	7e -Side	SER428-Side	0.54%
6	SER428-Side	7e -Side	1.16%

Notably, residues SER429, GLU321, HSD432, and SER428 exhibit a pronounced propensity to engage in hydrogen bonding with the ligand’s side atoms. This examination into hydrogen bond dynamics reveals not only the system’s stability nuances but also provides insights into the complex dance between the ligand and protein, deepening our grasp on the underlying forces dictating their synergy.

## Conclusion

In conclusion, the study comprehensively examined the electronic properties and reactivity of the synthesized compounds through Density Functional Theory (DFT) calculations. The HOMO-LUMO energy band gap analysis demonstrated its pivotal role in assessing molecule stability and reactivity. SwissADME evaluations highlighted compounds **7a**, **7c**, and **7d** for their favorable physicochemical properties, including solubility and drug-likeness. Molecular docking exhibited the strong binding affinities of **7f** and **7e** derivatives with intestinal alkaline phosphatase (IAP), further supported by Molecular Dynamics (MD) simulations and particularly in-vitro analysis. The MD simulations showcased the stability of protein-ligand complexes, as indicated by Root Mean Square Deviation (RMSD) and Root Mean Square Fluctuation (RMSF) analyses. Noteworthy hydrogen bond interactions were identified between specific IAP residues and ligand atoms, elucidating the structural basis of binding. Compound **7e** exhibited substantial inhibitory activity (IC_50_ = 0.308 ± 0.065 µM), surpassing the standard inhibitor (L–Phenyl alanine IC_50_ = 19.2 ± 0.11 µM).

This comprehensive integration of computational and experimental approaches sheds light on the potential therapeutic applications of the synthesized compounds. The study underscores the importance of understanding electronic properties, binding mechanisms, and structure-activity relationships in drug discovery and design. By providing a detailed investigation of these aspects, this research opens avenues for the development of novel pharmacologically active compounds with diverse applications.

### Electronic supplementary material

Below is the link to the electronic supplementary material.

Supplementary Material 1

## Data Availability

All the associated data will be available on request from corresponding author.

## References

[1] Sharma U, Pal D, Prasad R (2014). Alkaline phosphatase: an overview. Indian J Clin Biochem.

[2] Kaplan MM (1972). Alkaline phosphatase. Gastroenterology.

[3] Yang X, et al. Hepatic toxicity biomarkers. Biomarkers in toxicology. Elsevier; 2014. pp. 241–59. 10.1016/B978-0-12-404630-6.00013-0]

[4] Sebastián-Serrano Á (2015). Tissue-nonspecific alkaline phosphatase regulates purinergic transmission in the central nervous system during development and disease. Comput Struct Biotechnol J.

[5] Mornet E (2001). Structural evidence for a functional role of human tissue nonspecific alkaline phosphatase in bone mineralization. J Biol Chem.

[6] Bilski J, et al. The role of intestinal alkaline phosphatase in inflammatory disorders of gastrointestinal tract. Mediat Inflamm. 2017;2017. 10.1155/2017/9074601 10.1155/2017/9074601 PMC5339520 28316376

[7] Ghosh SS (2021). Over-expression of intestinal alkaline phosphatase attenuates atherosclerosis. Circul Res.

[8] Fawley J, Gourlay DM (2016). Intestinal alkaline phosphatase: a summary of its role in clinical disease. J Surg Res.

[9] Kühn F (2021). Targeting the intestinal barrier to prevent gut-derived inflammation and disease: a role for intestinal alkaline phosphatase. Visc Med.

[10] Banerjee S, et al. A critical review of benzimidazole: Sky-high objectives towards the lead molecule to predict the future in medicinal chemistry. Results Chem. 2023;101013. 10.1016/j.rechem.2023.101013]

[11] Zhuang C (2017). Chalcone: a privileged structure in medicinal chemistry. Chem Rev.

[12] Ibrahim AG (2022). New Thiadiazole modified chitosan derivative to control the growth of human pathogenic microbes and cancer cell lines. Sci Rep.

[13] Mohammed LA, Review on Benzimidazole Heterocyclic Compounds (2023). Synthesis and their Medicinal Activity Applications. SynOpen.

[14] Asif M, Husain A (2013). Analgesic, anti-inflammatory, and antiplatelet profile of hydrazones containing synthetic molecules. J Appl Chem.

[15] Gunasekar R (2022). Recent developments in enantio-and diastereoselective hydrogenation of N-heteroaromatic compounds. Org Biomol Chem.

[16] Thanusu J, Kanagarajan V, Gopalakrishnan M (2010). Spectral characterization of novel bis heterocycles comprising both piperidine and thiohydantoin nuclei. Res Chem Intermed.

[17] Yüce AO (2016). Experimental and quantum chemical studies on corrosion inhibition effect of 5, 5 diphenyl 2-thiohydantoin on mild steel in HCl solution. J Mol Liq.

[18] Tompkins JE (1986). 5, 5-Diaryl-2-thiohydantoins and 5, 5-diaryl N3-substituted 2-thiohydantoins as potential hypolipidemic agents. J Med Chem.

[19] Al-Obaid A (1996). 5-substituted-2-thiohydantoin analogs as a novel class of antitumor agents. Anticancer Drugs.

[20] Westerfeld WW, Marx JV, Richert DA (1970). Peripheral inhibition of thyroxine by thiohydantoins derived from amino acids. J Med Chem.

[21] Buchynskyy A (2017). 1-Benzyl-3-aryl-2-thiohydantoin derivatives as new anti-trypanosoma brucei agents: SAR and in vivo efficacy. ACS Med Chem Lett.

[22] El-Barbary AA (1994). S-Glucosylated hydantoins as new antiviral agents. J Med Chem.

[23] Chérouvrier J-R, Carreaux F, Bazureau JP (2004). Reactivity of 2-Thiohydantoins towards various Electrophilic reagents: applications to the synthesis of New 2-Ylidene-3, 5-dihydro-i>4 H-imidazol-4-ones. Molecules.

[24] Archer S, Unser MJ, Froelich E (1956). Some 5-(oxoalkyl)-2-thiohydantoins and their derivatives. J Am Chem Soc.

[25] Marton J (1993). Preparation and fungicidal activity of 5-substituted hydantoins and their 2-thio analogs. J Agric Food Chem.

[26] Kumar V (2012). Novel and efficient protocol for the syntheses of N-1 substituted thiohydantoin and a bicyclothiohydantoin under solvent-free conditions. Tetrahedron Lett.

[27] Han J (2011). Synthesis and herbicidal activity of 5-(4-hydroxybenzyl)-2-thioxoimidazolidin-4-one esters. Molecules.

[28] Zuo M (2017). Design and synthesis of indoline thiohydantoin derivatives based on enzalutamide as antiproliferative agents against prostate cancer. Eur J Med Chem.

[29] Mendgen T, Steuer C, Klein CD (2012). Privileged scaffolds or promiscuous binders: a comparative study on rhodanines and related heterocycles in medicinal chemistry. J Med Chem.

[30] Tahir T (2021). Pyridine scaffolds, phenols and derivatives of azo moiety: current therapeutic perspectives. Molecules.

[31] Abbasi M (2021). Synthesis, in Vitro, and in Silico studies of N-(Substituted-Phenyl)-3-(4-Phenyl-1-Piperazinyl) propanamides as potent alkaline phosphatase inhibitors. Russ J Bioorg Chem.

[32] Saeed A (2018). Synthesis of sulfadiazinyl acyl/aryl thiourea derivatives as calf intestinal alkaline phosphatase inhibitors, pharmacokinetic properties, lead optimization, Lineweaver-Burk plot evaluation and binding analysis. Bioorg Med Chem.

[33] Berkman SJ, Roscoe EM, Bourret JC (2019). Comparing self-directed methods for training staff to create graphs using Graphpad prism. J Appl Behav Anal.

[34] Bhavani K, Renuga S, Muthu S (2015). Quantum mechanical study and spectroscopic (FT-IR, FT-Raman, 13 C, 1H) study, first order hyperpolarizability, NBO analysis, HOMO and LUMO analysis of 2-acetoxybenzoic acid by density functional methods. Spectrochim Acta Part A Mol Biomol Spectrosc.

[35] Vanitha U (2021). Design, synthesis, characterization, molecular docking and computational studies of 3-phenyl-2-thioxoimidazolidin-4-one derivatives. J Mol Struct.

[36] Dennington R, Keith TA, Millam JM (2016). GaussView 6.0. 16.

[37] Daina A, Zoete V (2019). Application of the SwissDrugDesign online resources in virtual screening. Int J Mol Sci.

[38] Jia C-Y (2020). A drug-likeness toolbox facilitates ADMET study in drug discovery. Drug Discovery Today.

[39] Lipinski CA (1997). Experimental and computational approaches to estimate solubility and permeability in drug discovery and development settings. Adv Drug Deliv Rev.

[40] Al-Rashida M (2015). Diarylsulfonamides and their bioisosteres as dual inhibitors of alkaline phosphatase and carbonic anhydrase: structure activity relationship and molecular modelling studies. Bioorg Med Chem.

[41] Goodsell DS (2021). The AutoDock suite at 30. Protein Sci.

[42] Brown T (2014). ChemDraw Sci Teacher.

[43] Amir M (2020). Virtual high-throughput screening of natural compounds in-search of potential inhibitors for protection of telomeres 1 (POT1). J Biomol Struct Dynamics.

[44] Lee J (2016). CHARMM-GUI input generator for NAMD, GROMACS, AMBER, OpenMM, and CHARMM/OpenMM simulations using the CHARMM36 additive force field. Biophys J.

[45] Hansson T, Oostenbrink C, van Gunsteren W (2002). Molecular dynamics simulations. Curr Opin Struct Biol.

