# Supplementary material for: Exploration of newly synthesized azo-thiohydantoins as the potential alkaline phosphatase inhibitors via advanced biochemical characterization and molecular modeling approaches
Source: BMC Chem. 2024 Mar 6;18(1):47. doi: 10.1186/s13065-024-01149-8 (PMC10919040; doi:10.1186/s13065-024-01149-8)
Supplement: Supplementary file 1 — Supplementary Material 1 [file 13065_2024_1149_MOESM1_ESM.doc]

**Exploration of Newly Synthesized Azo-Thiohydantoins as the Potential Alkaline phosphatase Inhibitors via Advanced Biochemical Characterization and Molecular Modeling Approaches**

Hafiz Muhammad Attaullah^a^, Syeda Abida Ejaz^a*^, Pervaiz Ali Channar^b*^, Aamer Saeed^c*^, Rabail Ujan^d^, Seema Zargar^e^, Sajid Ali Channar^d^, Reshma Sahito^f^**,** Tanveer A. Wani^g^, Qamar Abbas^h,i^

*^a^Department of Pharmaceutical Chemistry, The Islamia University of Bahawalpur, Bahawalpur, 63100, Pakistan.*

*^b^Department of Basic Sciences and Humanities, Faculty of Information Science and Humanities, Dawood University of Engineering and Technology, Karachi 74800, Pakistan*

*^c^Department of Chemistry, Quaid-I-Azam University, Islamabad 45320, Pakistan*

*^d^Dr. M. A. Kazi Institute of Chemistry, University of Sindh, Jamshoro, Pakistan*

*^e^Department of Biochemistry, College of Science, King Saud University, P.O.Box 22452, Riyadh 11451, Saudi Arabia.^f^Department of Zoology University of Sindh, Jamshoro, Pakistan*

*^g^Department of Pharmaceutical Chemistry, College of Pharmacy, King Saud University, P.O.Box 22452, Riyadh 11451, Saudi Arabia.*

*^h^Department of Biology, College of Science, University of Bahrain, Sakhir, 32038 Kingdom of Bahrain*

*^i^College of Natural Sciences, Department of Biological Sciences, Kongju National University, Gongju-32588, Republic of Korea.*

*** Correspondence Author**

Syeda Abida Ejaz, Department of Pharmaceutical Chemistry, Faculty of Pharmacy, The Islamia University of Bahawalpur, Bahawalpur 63100, Pakistan. [abida.ejaz@iub.edu.pk](mailto:abida.ejaz@iub.edu.pk) Pervaiz Ali Channar, Department of Basic Sciences and Humanities, Faculty of Information Science and Humanities, Dawood University of Engineering and Technology, Karachi 74800, Pakistan. [pervaiz.ali@duet.edu.pk](mailto:pervaiz.ali@duet.edu.pk); Aamer Saeed, Department of Chemistry Quaid-i-Azam University-45320, Islamabad, Pakistan*.*  [aamersaeed@yahoo.com](mailto:aamersaeed@yahoo.com);

S**Table 1.** Physicochemical and pharmacokinetic properties for drug-likeness

| **Compounds** | **7a** | **7b** | **7c** | **7d** | **7e** | **7f** |
| --- | --- | --- | --- | --- | --- | --- |
| Formula | C16H13N5O2S | C16H13N5O5S2 | C16H11Cl2N5O2S | C17H14ClN5O2S | C17H13N5O4S | C16H12N6O4S |
| Molecular Weight g/mol | 339.37 | 419.43 | 408.26 | 387.84 | 383.38 | 384.37 |
| No. of Heavy atoms | 24 | 28 | 26 | 26 | 27 | 27 |
| No. of Aromatic heavy atoms | 12 | 12 | 12 | 12 | 12 | 12 |
| Fraction Csp3 | 0.06 | 0.06 | 0.06 | 0.12 | 0.06 | 0.06 |
| No. of Rotatable bonds | 4 | 5 | 4 | 4 | 5 | 5 |
| No. of H-bond acceptors | 5 | 8 | 5 | 5 | 7 | 7 |
| No. of H-bond donors | 2 | 3 | 2 | 2 | 3 | 2 |
| MR | 101.56 | 111.42 | 111.58 | 111.54 | 108.52 | 110.38 |
| TPSA | 121.74 | 184.49 | 121.74 | 121.74 | 159.04 | 167.56 |
| iLOGP | 2.53 | 1.18 | 2.69 | 2.98 | 2.09 | 2.23 |
| XLOGP3 | 2.79 | 1.54 | 4.05 | 3.78 | 2.32 | 2.62 |
| WLOGP | 2.1 | 2.42 | 3.4 | 3.06 | 1.79 | 2 |
| MLOGP | 0.88 | 0.29 | 1.9 | 1.63 | 0.57 | 0.03 |
| Silicos-IT Log P | 3.77 | 2.16 | 5.06 | 4.93 | 3.23 | 1.63 |
| Consensus Log P | 2.41 | 1.52 | 3.42 | 3.27 | 2 | 1.7 |
| ESOL Log S | -3.81 | -3.4 | -5 | -4.7 | -3.68 | -3.87 |
| ESOL Solubility (mg/ml) | 5.28E-02 | 1.68E-01 | 4.08E-03 | 7.68E-03 | 8.06E-02 | 5.15E-02 |
| ESOL Solubility (mol/l) | 1.56E-04 | 4.00E-04 | 9.99E-06 | 1.98E-05 | 2.10E-04 | 1.34E-04 |
| ESOL Class | Soluble | Soluble | Moderately soluble | Moderately soluble | Soluble | Soluble |
| Ali Log S | -5 | -5.02 | -6.31 | -6.03 | -5.3 | -5.79 |
| Ali Solubility (mg/ml) | 3.37E-03 | 3.97E-03 | 2.00E-04 | 3.62E-04 | 1.93E-03 | 6.25E-04 |
| Ali Solubility (mol/l) | 9.93E-06 | 9.47E-06 | 4.89E-07 | 9.33E-07 | 5.03E-06 | 1.63E-06 |
| Ali Class | Moderately soluble | Moderately soluble | Poorly soluble | Poorly soluble | Moderately soluble | Moderately soluble |
| Silicos-IT LogSw | -4.7 | -3.98 | -5.88 | -5.66 | -4.04 | -4.04 |
| Silicos-IT Solubility (mg/ml) | 6.83E-03 | 4.35E-02 | 5.44E-04 | 8.41E-04 | 3.51E-02 | 3.48E-02 |
| Silicos-IT Solubility (mol/l) | 2.01E-05 | 1.04E-04 | 1.33E-06 | 2.17E-06 | 9.16E-05 | 9.06E-05 |
| Silicos-IT class | Moderately soluble | Soluble | Moderately soluble | Moderately soluble | Moderately soluble | Moderately soluble |
| GI absorption | High | Low | High | High | Low | Low |
| BBB permeant | No | No | No | No | No | No |
| Pgp substrate | No | No | No | No | No | No |
| CYP1A2 inhibitor | Yes | No | Yes | Yes | Yes | Yes |
| CYP2C19 inhibitor | No | No | No | Yes | No | No |
| CYP2C9 inhibitor | Yes | No | Yes | Yes | No | Yes |
| CYP2D6 inhibitor | No | No | No | No | No | No |
| CYP3A4 inhibitor | Yes | No | Yes | Yes | Yes | Yes |
| log Kp (cm/s) | -6.39 | -7.77 | -5.91 | -5.98 | -6.99 | -6.78 |
| Lipinski violations | 0 | 0 | 0 | 0 | 0 | 0 |
| Ghose violations | 0 | 0 | 0 | 0 | 0 | 0 |
| Veber violations | 0 | 1 | 0 | 0 | 1 | 1 |
| Egan violations | 0 | 1 | 0 | 0 | 1 | 1 |
| Muegge violations | 0 | 1 | 0 | 0 | 1 | 1 |
| Bioavailability Score | 0.55 | 0.11 | 0.55 | 0.55 | 0.11 | 0.55 |
| PAINS alerts | 2 | 2 | 2 | 2 | 2 | 2 |
| Brenk alerts | 3 | 4 | 3 | 3 | 3 | 5 |
| Lead likeness violations | 0 | 1 | 2 | 2 | 1 | 1 |
| Synthetic Accessibility | 3.2 | 3.36 | 3.22 | 3.29 | 3.26 | 3.32 |


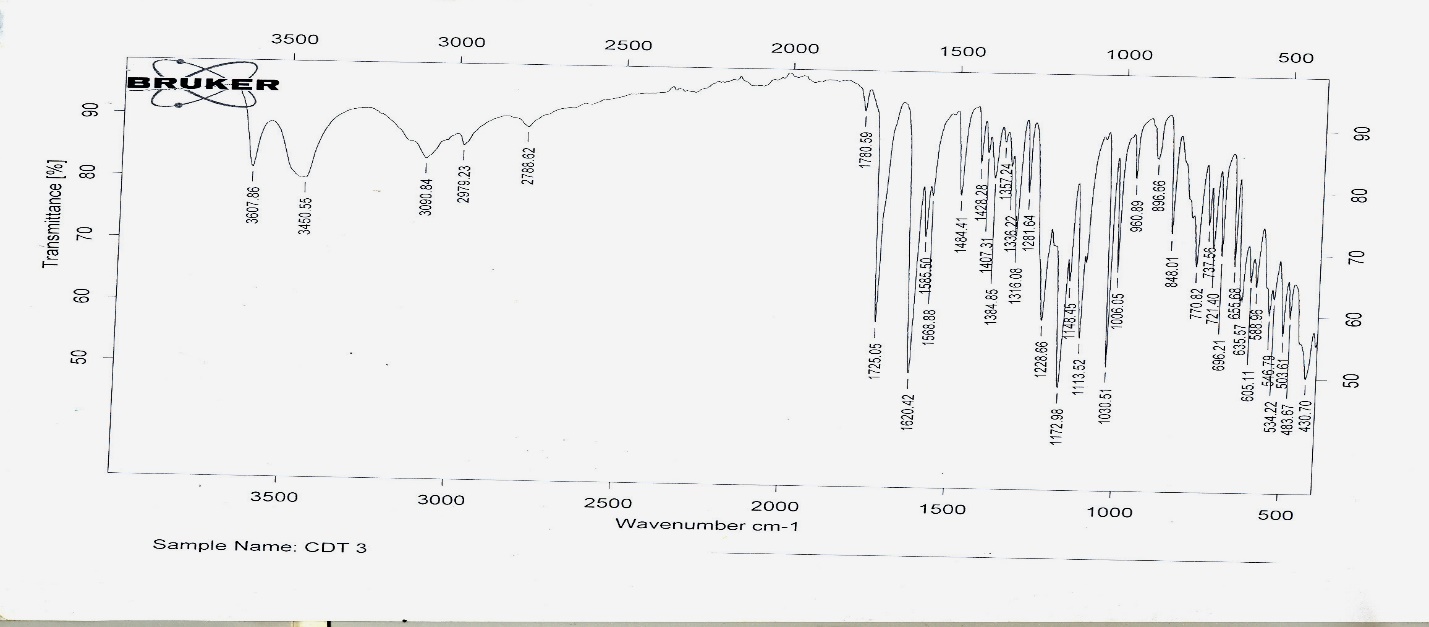


Figure S1. FTIR spectrum of synthesized derivative (7a)


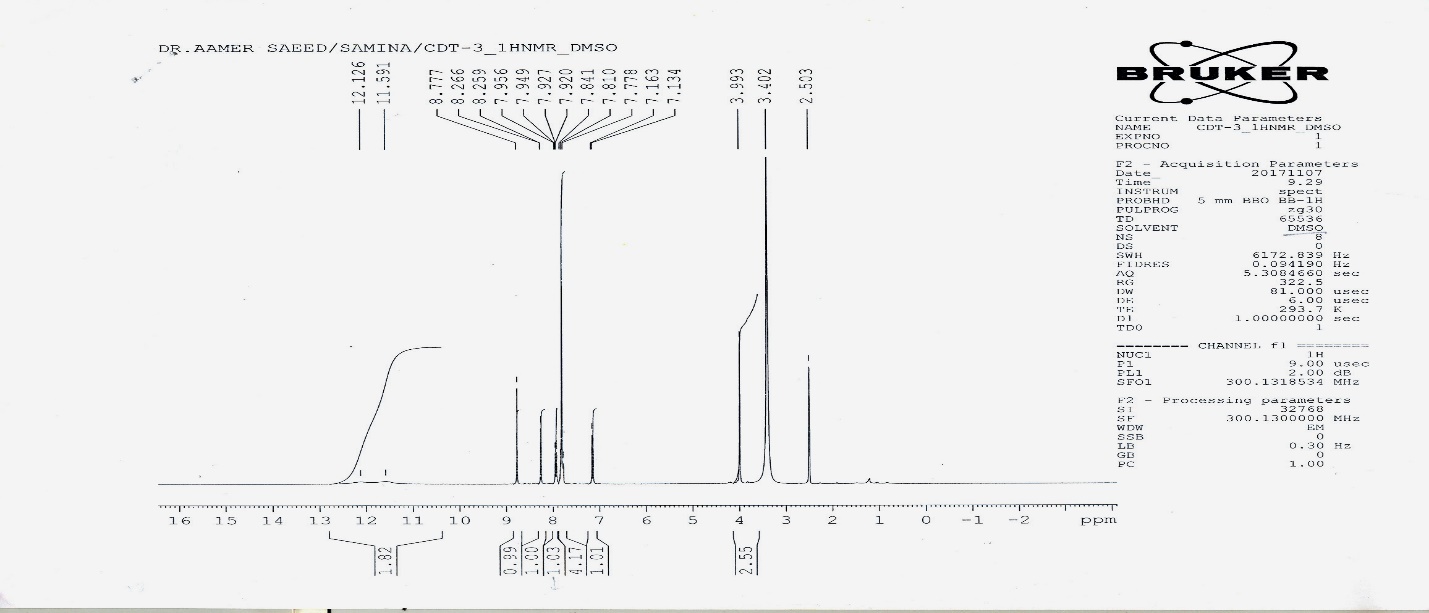


Figure S2.^1^H-NMR spectrum of synthesized derivative (7a)


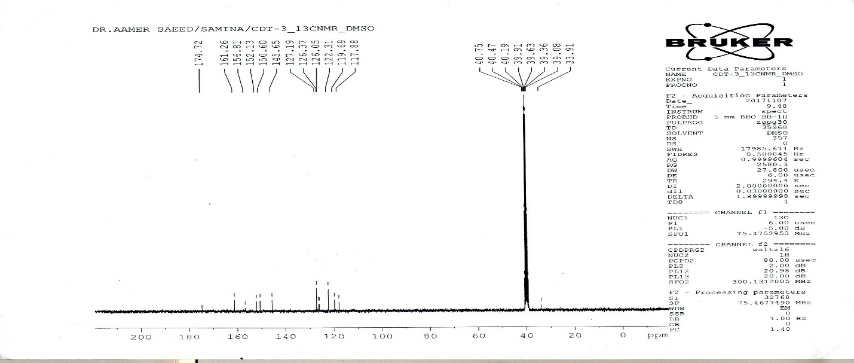


Figure S3. ^13^C-NMR spectrum of synthesized derivative (7a


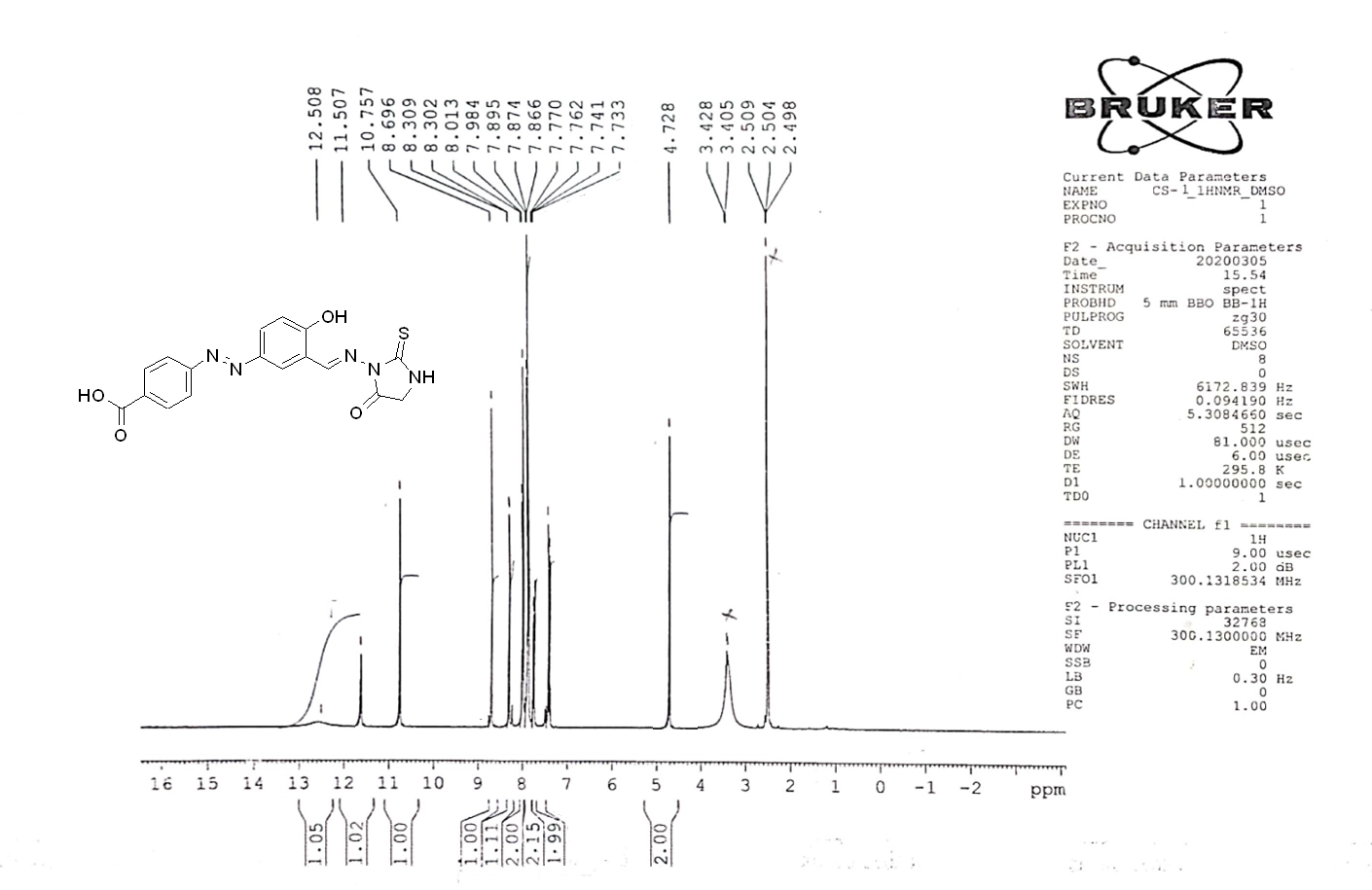


Figure S4.^1^H-NMR spectrum of synthesized derivative (7e)


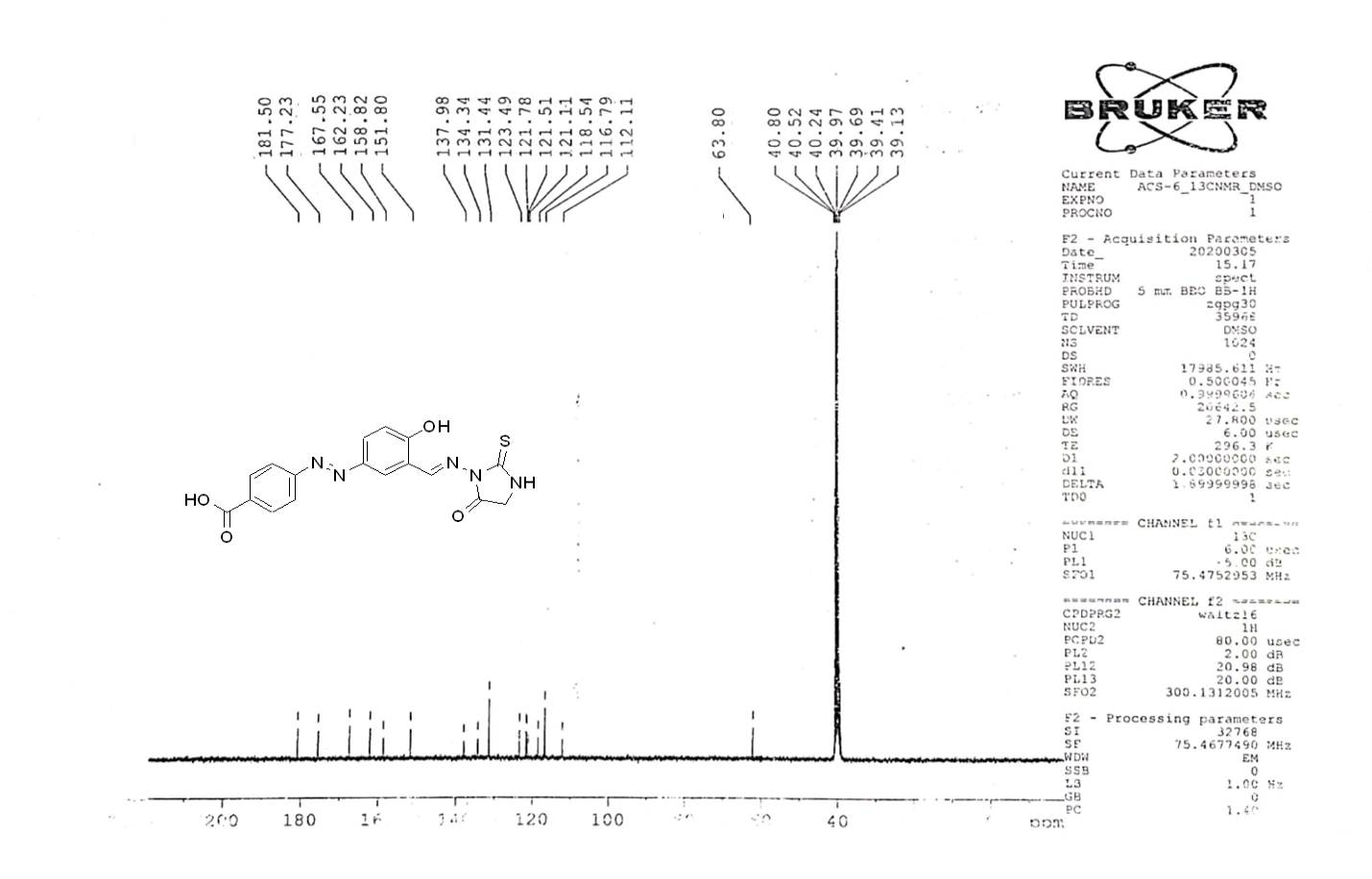


Figure S5. ^13^C-NMR spectrum of synthesized derivative (7e)


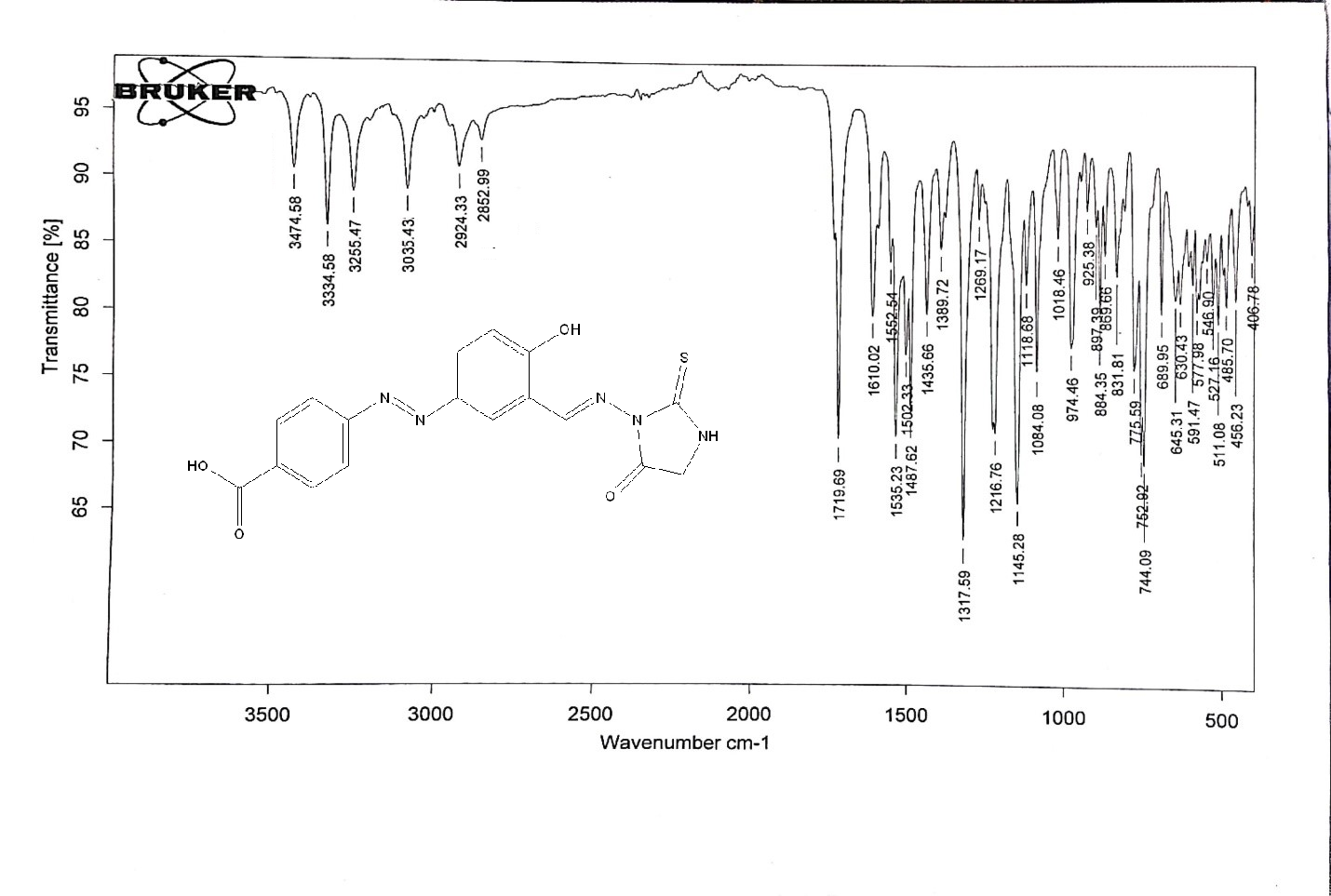


Figure S6. FTIR spectrum of synthesized derivative (7e)


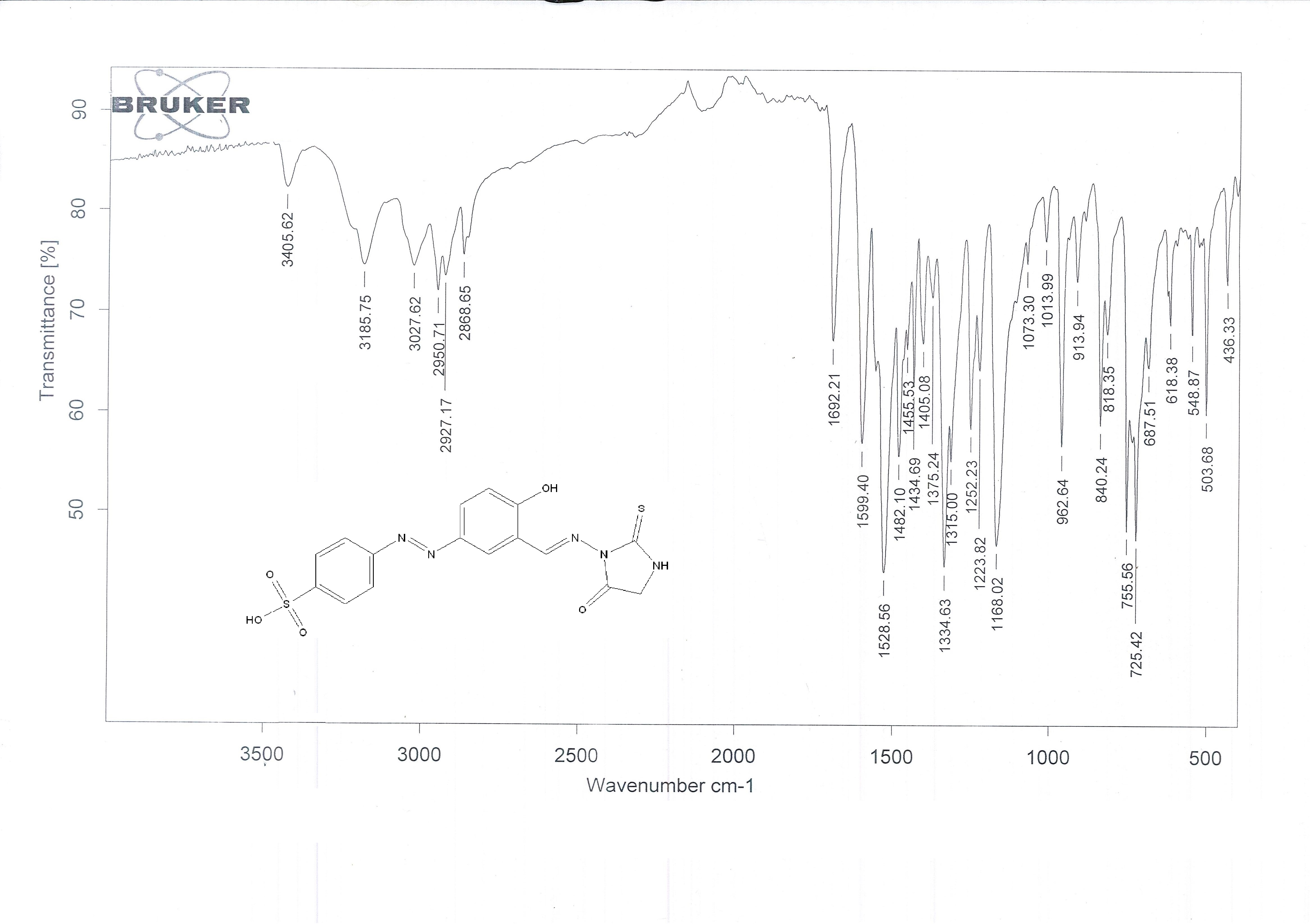


Figure S7. FTIR spectrum of synthesized derivative (7b)

Figure S8. FTIR spectrum of synthesized derivative (7f)

**
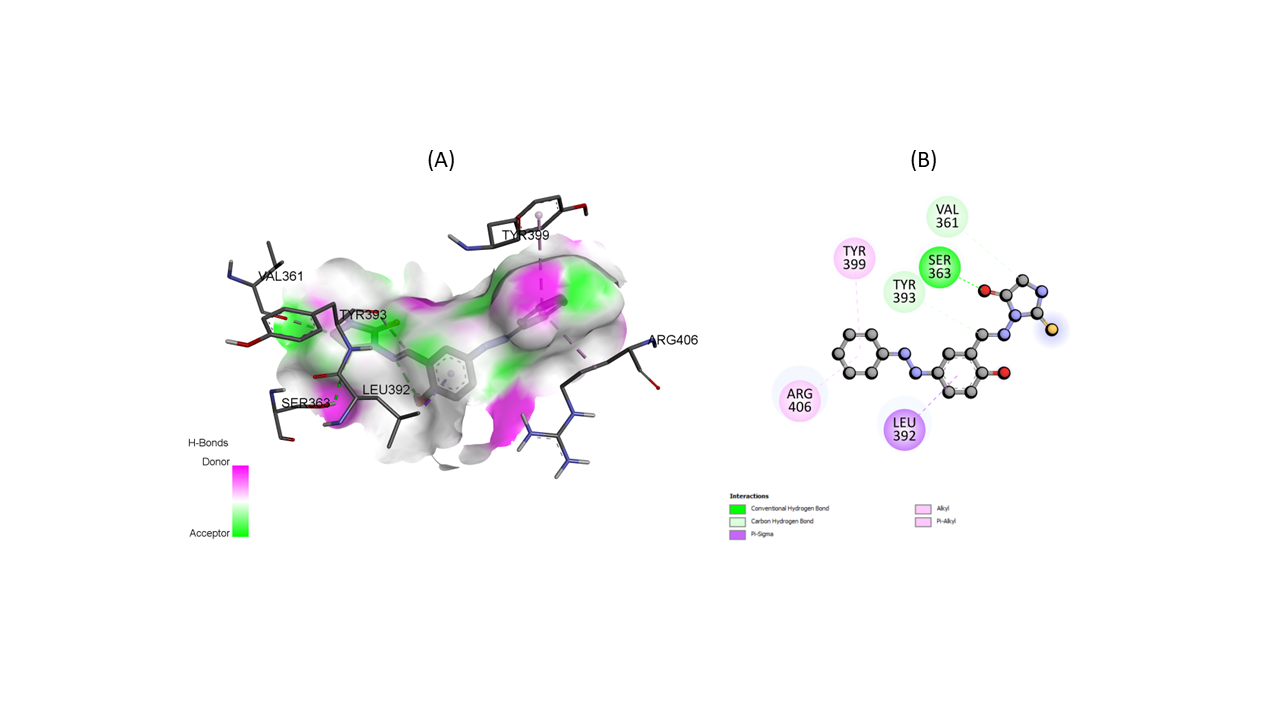
**

Figure S9.The predicted 2D and 3D binding pose of compound 7a within the active pocket of IAP


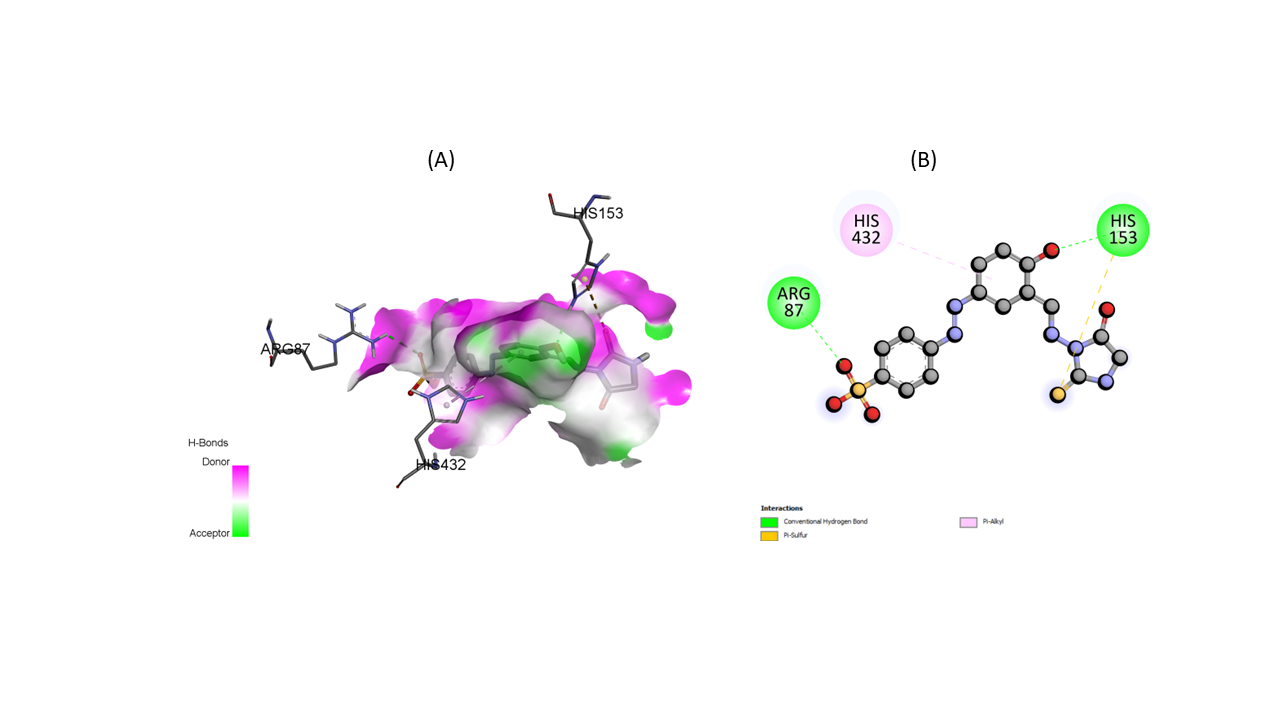


Figure S10.The predicted 2D and 3D binding pose of compound 7b within the active pocket of IAP


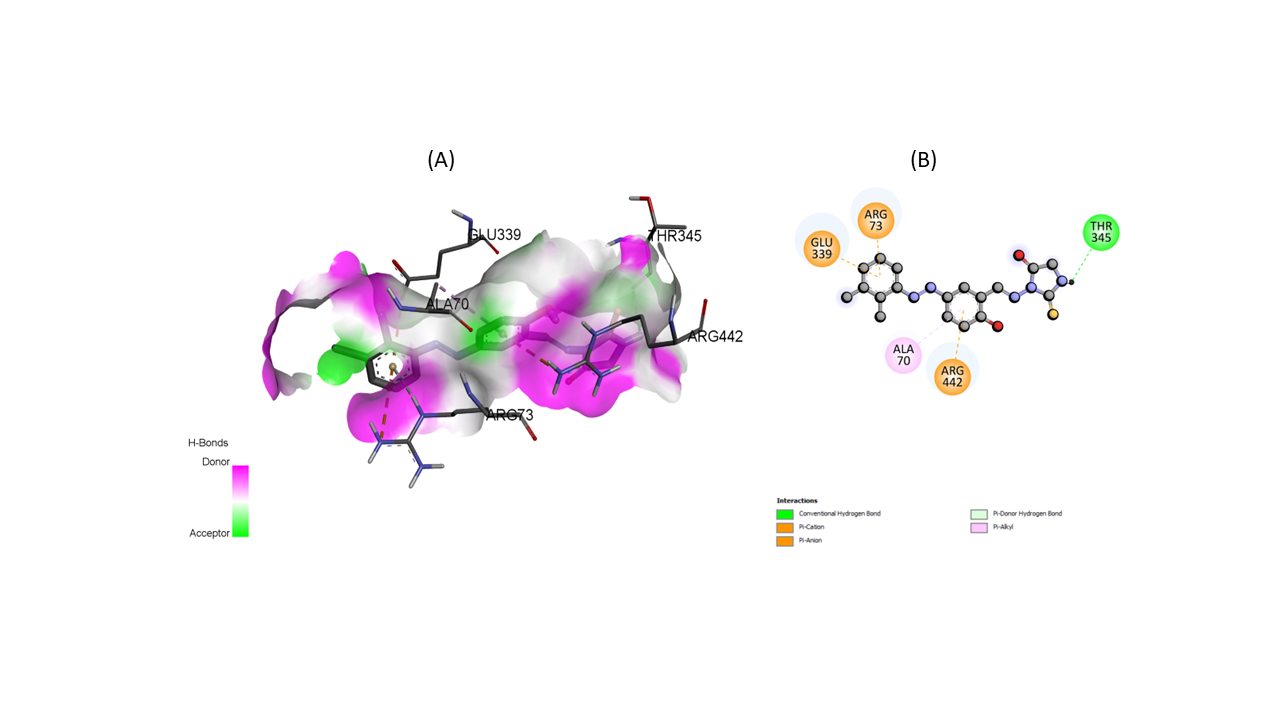


Figure S11.The predicted 2D and 3D binding pose of compound 7c within the active pocket of IAP


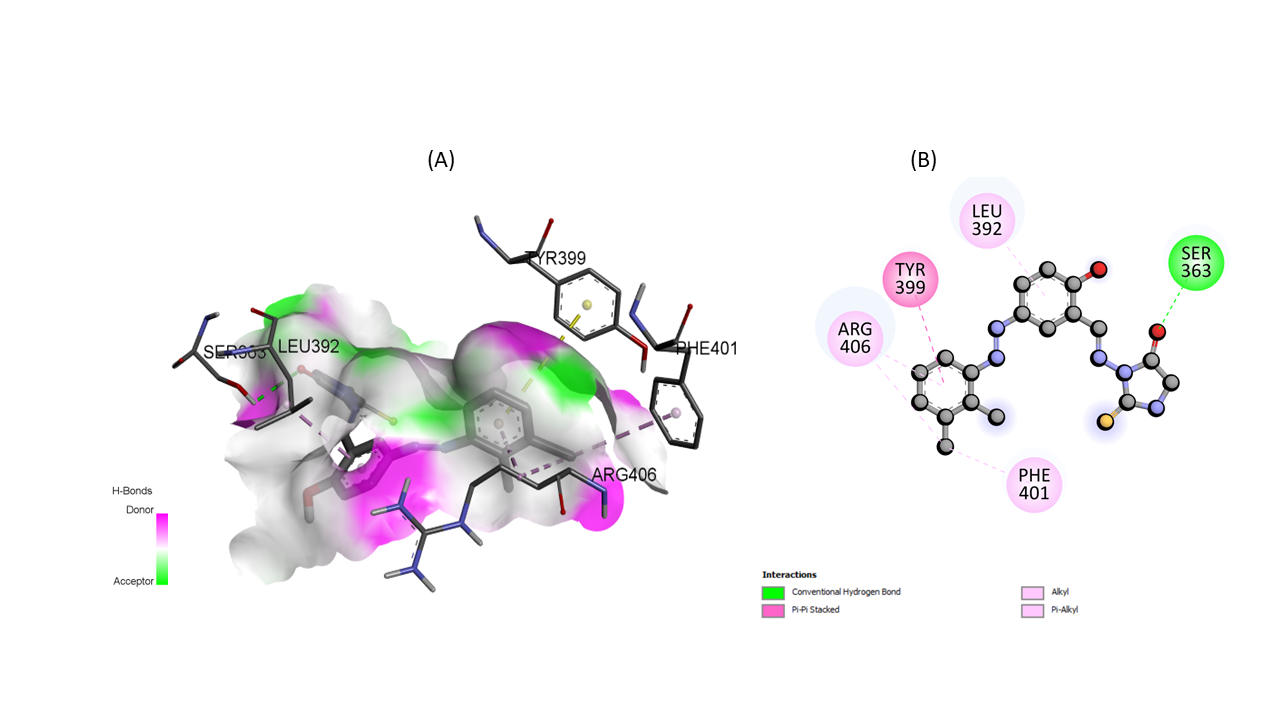


Figure S12.The predicted 2D and 3D binding pose of compound 7d within the active pocket of IAP
